# Host Hybridization Dominates over Cohabitation in Affecting Gut Microbiota of Intrageneric Hybrid *Takifugu* Pufferfish

**DOI:** 10.1128/msystems.01181-22

**Published:** 2023-02-23

**Authors:** Xingkun Jin, Haoyong Zhu, Yan Shi, Ziwei Chen, Yaohui Wang, Jian-Fang Gui, Zhe Zhao

**Affiliations:** a Jiangsu Province Engineering Research Center for Marine Bio-resources Sustainable Utilization, College of Oceanography, Hohai University, Nanjing, China; b State Key Laboratory of Freshwater Ecology and Biotechnology, Institute of Hydrobiology, The Innovation Academy of Seed Design, Chinese Academy of Sciences, Wuhan, China; c Jiangsu Zhongyang Group Co., Ltd., Nantong, China; University of Connecticut

**Keywords:** cohabitation, fish holobiont, gut microbiota, intrageneric hybrid, microbial transmission

## Abstract

Microbial symbionts are of great importance for macroscopic life, including fish, and both collectively comprise an integrated biological entity known as the holobiont. Yet little is known as to how the normal balance within the fish holobiont is maintained and how it responds to biotic and/or abiotic influences. Here, through amplicon profiling, the genealogical relationship between artificial F1 hybrid pufferfish with growth heterosis, produced from crossing female Takifugu obscurus with male Takifugu rubripes and its maternal halfsibling purebred, was well recapitulated by their gut microbial community similarities, indicating an evident parallelism between host phylogeny (hybridity) and microbiota relationships therein. Interestingly, modest yet significant fish growth promotion and gut microbiota alteration mediated by hybrid-purebred cohabitation were observed, in comparison with their respective monoculture cohorts that share common genetic makeups, implying a certain degree of environmental influences. Moreover, the underlying assemblage patterns of gut microbial communities were found associated with a trade-off between variable selection and dispersal limitation, which are plausibly driven by the augmented social interactions between hybrid and purebred cohabitants differing in behaviors. Results from this study not only can enrich, from a microbial perspective, the sophisticated understanding of complex and dynamic assemblage of the fish holobiont, but will also provide deeper insights into the ecophysiological factors imposed on the diversity-function relationships thereof. Our findings emphasize the intimate associations of gut microbiota in host genetics-environmental interactions and would have deeper practical implications for microbial contributions to optimize performance prediction and to improve the production of farmed fishes.

**IMPORTANCE** Microbial symbionts are of great importance for macroscopic life, including fish, and yet little is known as to how the normal balance within the fish holobiont is maintained and how it responds to the biotic and/or abiotic influences. Through gut microbiota profiling, we show that host intrageneric hybridization and cohabitation can impose a strong disturbance upon pufferfish gut microbiota. Moreover, marked alterations in the composition and function of gut microbiota in both hybrid and purebred pufferfish cohabitants were observed, which are potentially correlated with different metabolic priorities and behaviors between host genealogy. These results can enrich, from a microbial perspective, the sophisticated understanding of the complex and dynamic assemblage of the fish holobiont and would have deeper practical implications for microbial contributions to optimize performance prediction and to improve farmed fish production.

## INTRODUCTION

Microbial symbionts are of great importance for macroscopic life and contribute to multiple host biological processes, including metabolism, immunity, development, reproduction, and behavior, which has been proven using laboratory-reared gnotobiotic hosts (1, 2). Yet the complexity and dynamicity intrinsic in the microbial community have hampered researchers working to understand the mechanisms of the host-microbial interaction (3). Contemporary sequencing technologies with plummeting costs have been adopted widely to scrutinize the microbial and/or genetic properties conferring reciprocal associations with hosts, rendering it possible to systematically unravel the diversity-function relationship of the microbial community (4). Accumulating microbiota data have accelerated the renewal of concepts and fostered new research opportunities (1–3). The holobiont concept manifests both the integrated and multipartite nature of biological networks consisting collectively of a host and its associated microbiota, without prescribing any party to be a central attribute (1–3). Therefore, elucidating factors that underlie changes in either party is essential and can provide insights into how and to what extent the normal balance within holobiont (eubiosis) can be maintained and improved or even intervened and restored (rebiosis) if disrupted (dysbiosis) (4).

The animal gut microbiota is characterized as a prevailing microbial community colonizing a confined and contiguous habitat (i.e., gastrointestinal tract) that has distinct physicochemical properties harbored by diverse hosts (5). It has further implications for adopting general community ecological concepts of the macroorganism in understanding gut microbiota turnover and host-microbial interaction (6). Metacommunity theory has been applied broadly to elucidate microenvironmental drivers of microbiota succession (6). Specifically, an individual host’s gut is regarded as an interspersed habitat patch for microbes, which is connected by a common extrahost environment (5). Therefore, interhost proximity and interaction together raise the possibility for direct and/or indirect microbial transmissions and will contribute to microbial community composition (7, 8). Assemblage and turnover in ecological communities, including animal gut microbiota, are driven by two factors, namely, stochasticity and determinacy (6). Stochasticity indicates that each community member has an equal opportunity of occupying a given niche, wherein taxa occurrence and abundances are determined probabilistically by dispersal and drift (neutral effect) (9). Conversely, determinacy indicates that taxa presence-absence duality and abundance are governed by abiotic and/or biotic factors, which are associated with fitness difference determined by ecological filtering (niche effect) (10). Such filtering processes will leave tractable and predictable patterns reflected in community composition and diversity distribution (11). For instance, environmental filtering (determinacy) was the major ecological process governing the gut microbiota assembly of animal hosts, including fishes, and yet such contributions varied with host physiology, such as developmental stage and disease progression (12, 13).

Ray-finned fishes (Actinopterygii) comprise more than half of vertebrate diversity (14) and are also of vital economic significance, particularly in aquaculture (15). Fish growth traits attract great research interest in both fundamental and practical aspects and have long been known to be influenced by genetics-environmental interactions (16, 17). For instance, growth traits can be effectively selected in several fish lineages by different genetic approaches, such as artificial hybridization and transgenesis of growth factor genes (18–20). Wherein fast growth is usually ascribed to improved feed efficiency, that associated with altered digestive physiology reflected in such as enlarged surface area, increased fold number and evacuation rate of fish gastrointestinal tract (21, 22). Moreover, some prerequisites, including, among others, adequate feeding regime and sufficient feed ingredients, are also required (23, 24) to allow such host genetics-mediated growth promotion to occur (16, 17). As an indispensable part of the holobiont, the microbial response and/or modulation of gut microbiota in interactions between fish and environment should not be ignored, considering its interfacial roles involved in nutritional utilization, infection resistance, and ecological adaptation of the host (25). Hence, the outcomes of host genetics-environmental interactions not only contribute to the ecophysiological variations of fish but also will lead to perturbation in the diversity-function relationships of gut microbial communities thereof. Yet the mechanisms remain underexplored, and broad analyses of fish with variation in ecophysiology are particularly essential to obtain a generalized insight (26, 27).

*Takifugu* (aka pufferfish) possesses a bioaccumulation capacity for lethal tetrodotoxin (28) and has a compacted genome replete with shortened intergenic and intronic sequences devoid of repetitive elements (29), rendering it a distinctive model organism. Captive *Takifugu* becomes nontoxic under domestication and is consumed as a delicacy widely across Asian countries (30–32). Among others, two sister species, namely, Takifugu rubripes (31) and Takifugu obscurus (33), have the most promising traits of interest, which are fast growth and hypotonic tolerance, respectively. Aquaculture production of both species has increased progressively to satisfy a growing market demand (34, 35). Indeed, *T. obscurus* had been acclimated into complete freshwater residence (33), in contrast to *T. rubripes* that obligately resides in brackish water (36). Therefore, understanding how and determining the extent to which the growth performance can be improved and understanding how the environmental adaptability can be promoted have urgently become information needed to facilitate the rapid development of the pufferfish industry.

The gut microbiota contributes to the health and welfare of farmed fishes and can be regarded as an intermediate phenotype determined by both host genetics and environment, necessitating the incorporation of pairing the gut microbiota with host genetics for an assessment and prediction of trait performance (16, 37). Previously, we showed structurally and functionally different gut microbiota among farmed juvenile *T. obscurus* at an inconsistent growth rate (38). Recently, we obtained viable artificial F1 hybrids produced by crossing *T. obscurus* (♀) and *T. rubripes* (♂) (39). Using a hybrid *Takifugu* pufferfish model, this study attempts to address the following questions. (i) Will the host genetics, environment, and interactions between them impact host growth and gut microbiota, and to what extent will these impacts occur? According to holobiont concepts (1–3), we hypothesized that changes in genetics and culture modes of the host will influence trait performance and/or microbial assemblage within the pufferfish holobiont. To address this hypothesis, the F1 hybrids together with their maternal halfsibling *T. obscurus* purebreds were reared separately by genealogy (monoculture) or equal proportionally cohabitated (coculture), and the host body mass and gut microbiota profile were analyzed integratedly. (ii) What are the phenotypic and functional implications of the gut microbiota underlying host genetics-environmental interactions? The propensity for colonization and proliferation in the gut varies greatly across microbial taxa (40, 41), and as such, we hypothesized that the compositional and functional congruence of disturbed gut microbiota can be resolved. Accordingly, the bacterial phenotypes and predicted metagenomes of gut microbiota were inferred. (iii) What roles of different ecological processes govern the assemblage of the gut microbial community? Given the potential homogenizing effects (42) on the gut microbiota of cohabited fishes, we hypothesized that the interhost dissimilarity of the gut microbial community would be reduced while in cohabitation. To test this hypothesis, the patterns of ecological processes were discerned by the inference of phylogenetic signals and quantification of determinacy and stochasticity influence.

## RESULTS

### Synergistic effects of hybridization and cohabitation on fish growth: the host aspect of the pufferfish holobiont.

**FIG 1:**
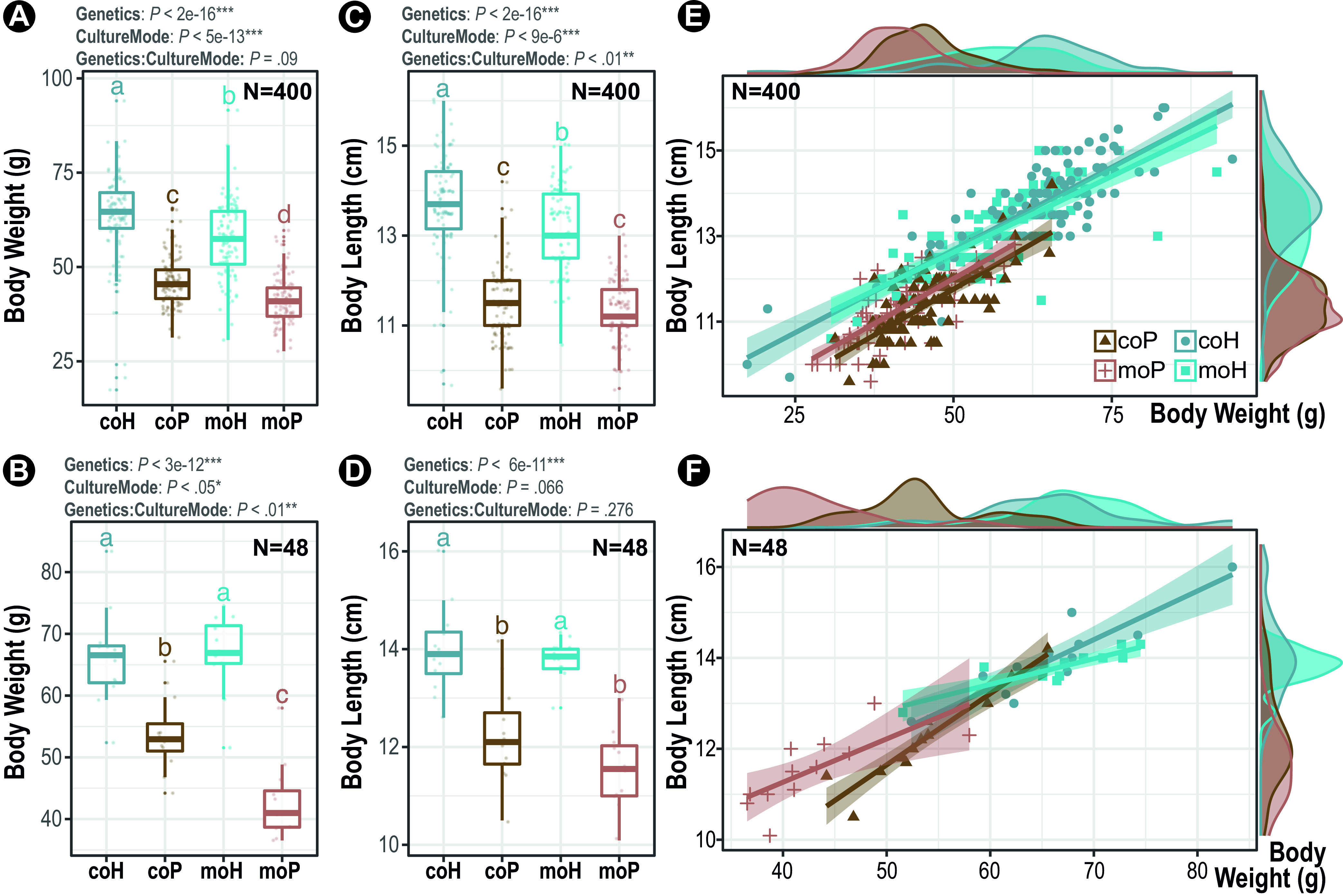
Explained variance in fish body mass by host intrageneric hybridity (hybrid [H] and purebred [P]) and culture modes (coculture [co] and monoculture [mo]). Boxplots denote differences of fish body weight across groups within the full data set (A) and subset (B) and fish body length within the full data set (C) and subset (D). Statistics are shown as the panel titles. In each box, the horizontal bold bar denotes medians, the box height denotes interquartile range (25th to 75th percentile), and the whiskers denote the value range within 1.5 times the interquartile. Lowercase letters denote statistical significance reported at a confidence level of 0.95. Shown are two-dimensional scatterplots of all (E) and a subset (F) pufferfish individuals based on body mass metrics, including weight and length. The marginal density plots show the distributions of indicated body mass metrics per fish group. Lines denote the linear regression of pufferfish body mass (Spearman’s rank).

The individual body weight and fork length were recorded to measure the explained variance in fish body mass by (i) host hybridity, i.e., hybrid (“H”) versus purebred (“P”); and by (ii) culture modes, i.e., coculture (“co”) versus monoculture (“mo”) (see Fig. S1A and Table S1 in the supplemental material), respectively. Of note, such an unambiguous grouping of fish individuals by hybridity, particularly for the cocultures, is achieved by visually distinguishing the fish skin color patterns. That is, hybrids produced by *T. obscurus* (♀) and *T. rubripes* (♂) tend to be darker with more shattered yellowish-white spots spread over the dorsal part as described previously (39, 43). First, we observed significantly larger body weight in hybrids than that of their maternal halfsibling purebreds in both “full” data sets with a sum of 400 pufferfishes (aligned rank transformed [ART] two-way analysis of variance [ANOVA], *P *< 2.2e-16) (Fig. 1A) and sub ones with 48 randomly selected individuals for downstream gut microbiota profiling (two-way ANOVA, *P *= 3.0e-12) (Fig. 1B). Similar trends held true for the fork length in both full data sets (ART two-way ANOVA, *P *< 2.2e-16) (Fig. 1C) and sub ones (two-way ANOVA, *P *= 6.4e-11) (Fig. 1D). These results indicated an evident growth vigor driven by host hybridization. In addition, significantly increased body weight and, to a lesser extent, fork length were observed in hybrid-purebred cohabitants compared with those of their respective monocultures that differed in genetic background for both full data sets (“weight,” *P *= 5.5e-13; “length,” *P *= 8.9e-6; ART two-way ANOVA) and sub ones (weight, *P *< 0.05; length, *P *= 0.066; two-way ANOVA), indicating an effect of cohabitation on pufferfish growth performance. Importantly, significant interactions between above two explanatory variables were also observed in both full data sets (length, *P *< 0.01; ART two-way ANOVA) and sub ones (weight, *P *< 0.01; two-way ANOVA), indicating a synergistic effect between host genetics and culture mode that affected fish growth.

10.1128/msystems.01181-22.1 FIG S1 Experimental design and evaluation of the sequencing depth of microbiota across sample groups. (A) The breeding design and distribution of progenies among respective rearing tanks. (“TaRu,” *T. rubripes*; “TaOb,” *T. obscurus*) (B) Rarefaction curves of detected microbial taxa (observed ESVs) within each indicated sample tend to be “flat” as the numbers of sampling increase, indicating the sampling covers the most microbial taxa (saturated). (C) Rarefaction curves of detected microbial taxa (numbers of observed ESVs) in each indicated group. Vertical bars denote standard error. Sample size for each indicated groups are as follows: purebred (*n* = 24), including monoculture (moP; *n* = 12) and coculture (coP; *n* = 12); hybrid (*n* = 24), including monoculture (moH; *n* = 12) and coculture (coH; *n* = 12); and feed (Fd; *n* = 3). Download FIG S1, PDF file, 0.2 MB. Copyright © 2023 Jin et al. 2023 Jin et al. https://creativecommons.org/licenses/by/4.0/ This content is distributed under the terms of the Creative Commons Attribution 4.0 International license.

10.1128/msystems.01181-22.10 TABLE S1 Metadata. Download Table S1, PDF file, 0.04 MB. Copyright © 2023 Jin et al. 2023 Jin et al. https://creativecommons.org/licenses/by/4.0/ This content is distributed under the terms of the Creative Commons Attribution 4.0 International license.

Furthermore, an individual pufferfish was clustered on the basis of its aforementioned body mass measures by principal-component analysis (PCA) to obtain a combined metric of fish growth traits. Also, the quality of representation (cos^2^) for both body mass measures was equally greater than 90% for the PC1 in either full data sets or sub ones. The per-individual PC1 (Table S1) was used as a body mass proxy for downstream analysis. To scrutinize and visualize individuals characterized by body mass, the results of univariate analyses of weight and length, respectively, were visualized by scatterplots, and all pufferfishes were well separated by both variables in full data sets (Fig. 1E) and sub ones (Fig. 1F).

### Gut microbiota responses to host hybridization and cohabitation: the microbial aspect of pufferfish holobiont.

Here, the microbiota of pufferfish gut and feed were profiled by 16S rRNA amplicon sequencing. A total of 6.4 million reads with an average length of 400 bp from 51 samples (a subset of 48 from 400 individuals; 3 technical feed replicates) were obtained. Through USEARCH-based read filtering and chimera discarding, followed by Ribosomal Database Project (RDP) classifier-based chloroplast sequence filtering, a total of 5,192 exact sequence variants (ESVs) were clustered. A sum of 5.8 million (90.7%) high-quality reads were retained (mappable to ESVs) with an average 113,561 reads per sample (Table S1). The coverage rate was estimated on rarefied data sets at 10,000 sequences per sample using rarefaction curves, which thereby ensured an adequate sequencing depth, and we retained all samples for further analysis (Fig. S1B and C).

**FIG 2:**
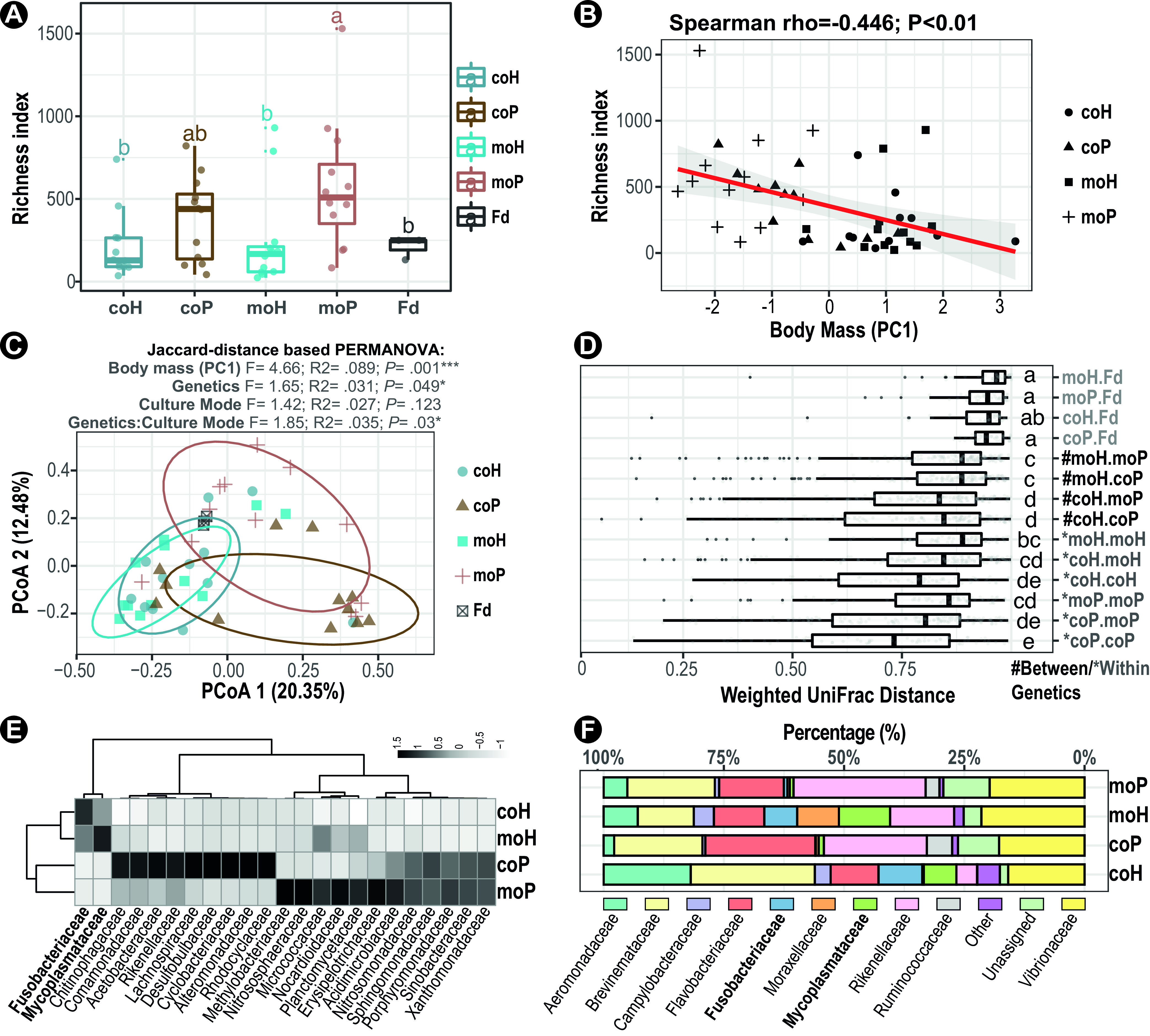
Associations of host hybridity and cultured modes with gut microbiota. (A) Comparisons of alpha diversity (Species richness) across the gut microbiota of different sources (“Fd” denotes feed). (B) Anticorrelations between the alpha diversity of pufferfish gut microbiota and the body mass index. The red line denotes the linear regression of alpha diversity and pufferfish body mass (Spearman’s rank), and statistics are showed in the panel title. (C) Two-dimensional scatterplot of all gut microbiota based on principal-coordinate analysis (PCoA) on Jaccard distance. Percentage of explained variance and statistics (PERMANOVA test with 999 permutations) were shown in the panel title. A normal data ellipse was drawn at a confidence level of 0.68 for each group. (D) Comparisons of paired dissimilarities (weighted UniFrac distances). The gut-feed pairs were labeled in light gray; the between- and within-genetics pairs were labeled in black and dark gray, respectively. (E) Featured bacterial families discriminating gut microbial community groups were identified by Random-Forest. Bacterial taxa were hierarchically clustered according to classification importance per taxa (column) in discriminating a given gut microbial community (row) from others. (F) Relative abundance of top-10 bacterial families across the gut microbiota of different pufferfish groups. Taxa which have a lower relative abundance were grouped into “other.”

The calculated indices of metataxonomic diversity, including richness (observed ESVs), Chao1, Shannon, and Simpson (see Fig. S2 in the supplemental material), all suggested that the alpha diversity of the gut microbial community was markedly higher in purebreds (moP) than that in their maternal halfsibling hybrids (moH) in monoculture, and such a difference was slightly yet significantly diminished in hybrid-purebred cohabitants (coP versus coH) (Fig. 2A). In addition, an anticorrelation between the alpha diversity and the fish body mass was observed (weight rho = −0.45, *P* < 0.01; length rho = −0.46, *P* < 0.001) (Fig. 2B). While, for each fish group separated by both genetics and culture modes, such an anticorrelation could be observed only in coculture purebred pufferfishes (length rho = −0.73, *P *< 0.01; weight rho = −0.69, *P *< 0.05) (Fig. 2B). Furthermore, comparisons of intercommunity dissimilarities (beta diversity) based on Jaccard distance showed that host body mass explained a larger fraction of variance (permutational multivariate analysis of variance [PERMANOVA], F = 4.66; R^2^ = 0.089; *P* = 0.001), followed by host genetics (F = 1.65; R^2^ = 0.031; *P *= 0.049) (Fig. 2C). Although the effect of culture mode alone was not statistically significant (F = 1.42; R^2^ = 0.027; *P *= 0.123), its interactions with the host genetics was significant (F = 1.85; R^2^ = 0.035; *P *= 0.03) (Fig. 2C). The permutational analysis of multivariate dispersion (PERMDISP) results showed that the intergroup dispersions between either genetics or culture modes were not significantly different in homogeneity (Permutest, *P *> 0.05) (see Fig. S3 in the supplemental material). Moreover, comparisons of weighted UniFrac distances showed that fish gut had greater dissimilarities to feed than those of all interhost pairs (*P *< 0.05; Mann-Whitney U test). Among others, the cocultured pufferfish of both genetics (coH and coP) had the shortest average intragroup distances and yet had the greatest dispersions (pairs “coH.coH” and “coP.coP”), suggesting a greater within-community similarity heterogeneity (Fig. 2D). Specifically, the gut microbiota was dramatically different compared with that of the feed, and it harbored less variation within genetics (asterisks in Fig. 2D) than between genetics (hashtags in Fig. 2D), which well recapitulated host genealogy. Furthermore, the discriminative taxa between fish groups were classified by the Random-Forest machine-learning approach, and a total of 23 bacterial families were identified to be able to classify a sample to group with 75% accuracy after 10-fold cross-validation (Fig. 2E). Wherein, two indicative bacterial families for hybrid pufferfish gut microbiota were identified, namely, *Mycoplasmataceae* (*Tenericutes*) and *Fusobacteriaceae* (*Fusobacteria*). Conversely, more different indicative families for purebred gut microbiota were characterized than the hybrid counterparts and brought into correspondence with the estimated higher alpha diversity of the formers. Consistently, the aforementioned two discriminative bacterial families were also found to be among the top-10 abundant taxa in hybrid pufferfish gut, by comparing the metataxonomic compositions of gut microbiota among fish groups (Fig. 2F; see Fig. S4 in the supplemental material). Besides, a large majority of predominant gut bacterial families were found to be shared among fish groups, including *Aeromonadaceae*, *Brevinemataceae*, *Flavobacteriaceae*, and *Vibrionaceae*, in contrast to the predominant *Enterobacteriaceae* (48.9%), *Moraxellaceae* (24.9%) and *Burkholderiaceae* (22.2%) present in feed microbiota.

10.1128/msystems.01181-22.2 FIG S2 Calculated alpha diversity based on indices of Chao1 (A), Shannon_e (logarithm base *e*) (B), and Simpson (C) among five groups of microbiota. For each box, the horizontal bold bar denotes medians, the height of the box denotes the interquartile range (25 to 75th percentile), and the whiskers mark the value range within 1.5 times the interquartile. Lowercase letters denote statistical significance reported by Mann-Whitney U test at a confidence level of 0.95. Download FIG S2, PDF file, 0.5 MB. Copyright © 2023 Jin et al. 2023 Jin et al. https://creativecommons.org/licenses/by/4.0/ This content is distributed under the terms of the Creative Commons Attribution 4.0 International license.

10.1128/msystems.01181-22.3 FIG S3 Evaluation of homogeneities of gut microbiota samples. (A) Principal-coordinate plot depicts the Jaccard dissimilarity distances among sample groups and its group centroids. (B) Boxplot of distances to the group centroid for the four groups of gut microbial communities. Download FIG S3, PDF file, 0.1 MB. Copyright © 2023 Jin et al. 2023 Jin et al. https://creativecommons.org/licenses/by/4.0/ This content is distributed under the terms of the Creative Commons Attribution 4.0 International license.

10.1128/msystems.01181-22.4 FIG S4 Stacked bars depict the relative abundance of top-10 bacterial phylums (A), classes (B), orders (C), and genus (D) across sample groups. The taxon ranks with lower relative abundance were grouped into “other.” Download FIG S4, PDF file, 0.2 MB. Copyright © 2023 Jin et al. 2023 Jin et al. https://creativecommons.org/licenses/by/4.0/ This content is distributed under the terms of the Creative Commons Attribution 4.0 International license.

### Phenotypic and functional implications of the gut microbiota underlying host genetics-environmental interactions.

**FIG 3:**
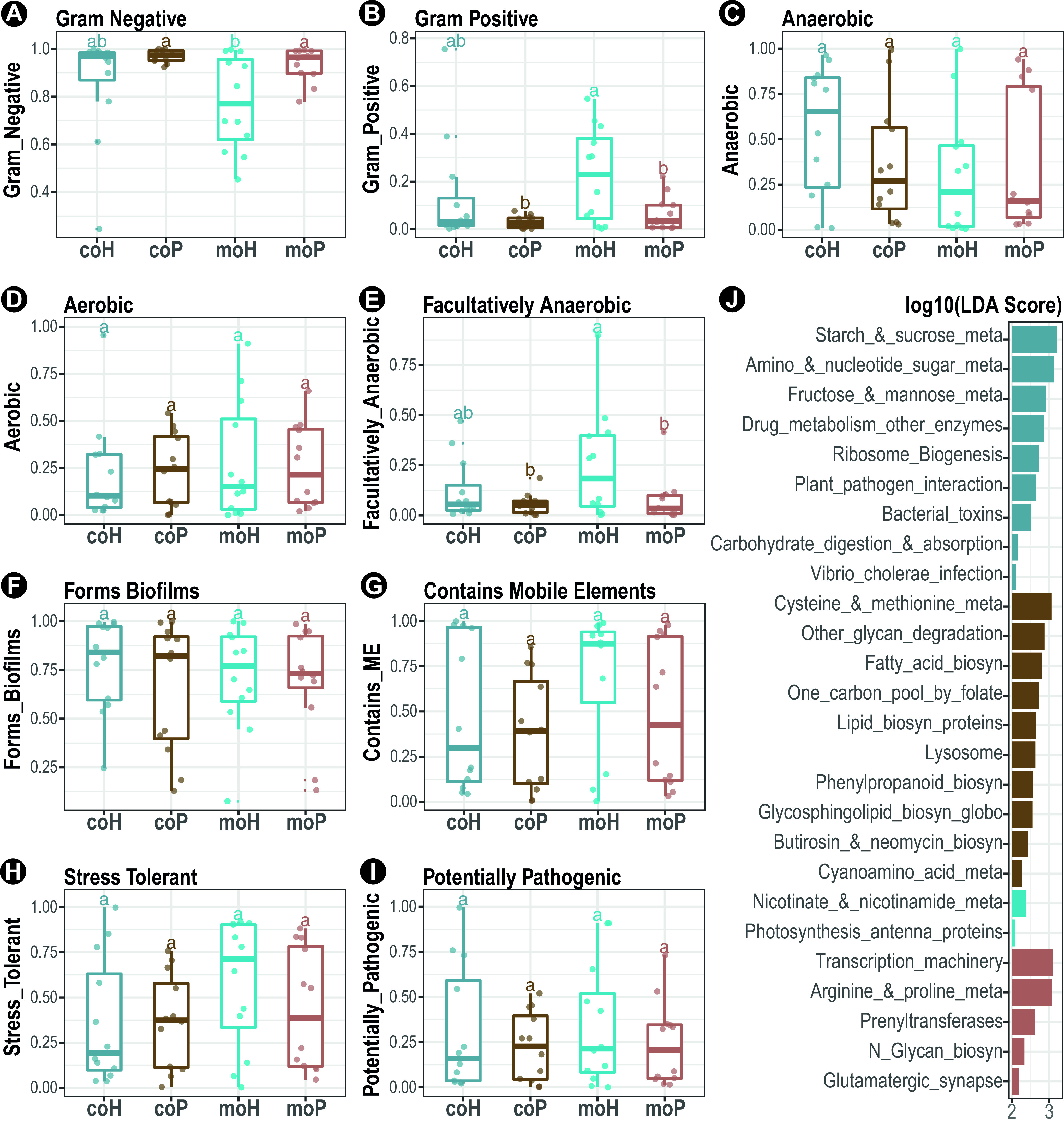
Inferred bacterial phenotypes and functions of pufferfish gut microbiota. The relative abundance of each indicated bacterial phenotype was inferred by BugBase and compared across different groups. Relative abundances of bacteria differing in Gram staining were shown for Gram-negative (A) and for Gram-positive (B) groups. Relative abundances of bacteria differing in oxygen tolerance phenotypes were shown for anaerobic (C), aerobic (D), and facultatively anaerobic (E) bacteria. Relative abundances of bacteria differing in latent pathogenicity phenotypes were shown for biofilm formation (F), for containing mobile elements (G), for oxidative stress tolerance (H), and for potentially pathogenic (I). Lowercase letters denote groups, and statistical significance was reported by pairwise Mann-Whitney U tests with false discovery rate correction at a confidence level of 0.95. (J) Bars denote the LEfSe-estimated discriminative KEGG pathways that are enriched in each indicated group. Bar width denotes the log_10_-transformed LDA effect size.

The organism-level phenotypes of bacterial communities, including Gram staining, oxygen tolerance, and pathogenic potential, were inferred by BugBase. The results showed that the gut microbiota of all fish groups are comprised of an overwhelming majority of Gram-negative bacteria (89%), which is significantly enriched in the purebred groups (95%) (Fig. 3A). Of note, the hybrid groups also had significantly nonnegligible portions of Gram-positive bacteria (18%), particularly in monocultures (23%) compared with those in the cocultures (14%) (Fig. 3B; see Fig. S5 in the supplemental material). Regarding the oxygen tolerance traits (Fig. 3C to E), the fish gut was predominated by anaerobes (39%), followed by aerobes (25%), and facultatively anaerobes (13%). While such proportions deviated slightly between fishes grouped by genetics, both anaerobes and facultative anaerobes were more present in the hybrid groups (42% and 19%, respectively) than those in the purebred ones (36% and 7%, respectively); albeit no statistical significance was found for the anaerobe phenotype (Fig. 3C). All fish groups were predicted to have prominent portions of bacteria that can form biofilms (71%), contain mobile elements (51%), and, to a lesser extent, be oxidative stress tolerant (44%) and potentially pathogenic (28%) (Fig. 3F to I).

10.1128/msystems.01181-22.5 FIG S5 Stacked bars depict the relative abundance of indicated bacterial families in contributing to the bacterial phenotypes that are inferred by BugBase. Download FIG S5, PDF file, 0.2 MB. Copyright © 2023 Jin et al. 2023 Jin et al. https://creativecommons.org/licenses/by/4.0/ This content is distributed under the terms of the Creative Commons Attribution 4.0 International license.

In addition, the bacterial families that were significantly correlated with aforementioned bacterial phenotypes were further scrutinized. For instance, the prominent Gram-negative bacteria were attributed to *Brevinemataceae* (19%; Rho = 0.5) and the Gram-positive ones were attributed to *Fusobacteriaceae* (4%; Rho = 0.5) and *Mycoplasmataceae* (5%; Rho = 0.3). The anaerobes, aerobes, and facultative anaerobes were attributed to *Brevinemataceae* (Rho = 0.8), *Aeromonadaceae* (8%; Rho = 0.4), and *Mycoplasmataceae* (Rho = 0.2), respectively. The biofilm-forming bacteria were attributed to *Brevinemataceae* (Rho = 0.5); and the mobile elements containing potentially pathogenic and oxidative stress-tolerant bacteria all were attributed mainly to *Aeromonadaceae* (Rho: 0.5~0.6). In addition, *Fusobacteriaceae* also contributed to the mobile elements containing the phenotype (Rho = 0.4) (see Fig. S6 in the supplemental material).

10.1128/msystems.01181-22.6 FIG S6 Heatmaps depict the correlations between the relative abundance of each indicated bacterial phenotype inferred by BugBase, the relative abundance of the top-20 abundant bacterial families, KEGG pathway categories, and body mass. Color scale denotes correlatedness, and only statistically significant (*P *< 0.05) Spearman’s Rho values were shown in each heatmap pixel. Download FIG S6, PDF file, 0.02 MB. Copyright © 2023 Jin et al. 2023 Jin et al. https://creativecommons.org/licenses/by/4.0/ This content is distributed under the terms of the Creative Commons Attribution 4.0 International license.

To gain further biological insights into the functional differences among the gut bacterial communities that varied by fish groups, the metagenomes (KEGG pathway-level categories) were predicted by phylogenetic investigation of communities by reconstruction of unobserved states (PICRUSt). The results showed that both fish body weight and length were positively correlated with the abundance of the “carbohydrate digestion and absorption” pathway (Rho = 0.2 and 0.3) (Fig. S6). Moreover, certain pathways related to carbohydrate and lipid metabolisms were enriched in the gut microbiota of cohabitants (coH and coP) compared with their respective monocultured counterparts (Fig. S6). Specifically, two pathways, including “starch and sucrose metabolism” and “amino sugar and nucleotide sugar metabolism,” with both being attributed to *Brevinemataceae* (Rho = 0.7 and 0.6), were enriched in the hybrid cohabitants (coH, log_10_ linear discriminant analysis [LDA] of >3) (Fig. 3J). Whereas, other pathways, including “cysteine and methionine metabolism” and “other glycan degradation,” which were attributed to *Brevinemataceae* (Rho = 0.6) and *Rikenellaceae* (Rho = 0.3), respectively, were enriched in the purebred cohabitants (coP, log_10_LDA of >2.5) (Fig. 3J). Regarding the monocultures, pathways, including “Nicotinate and nicotinamide metabolism” (metabolism of cofactors and vitamins) and “photosynthesis-antenna proteins” (energy metabolism), that were attributed to *Brevinemataceae* (Rho = 0.5) and some less abundant (average of <1.5%) bacterial families (e.g., *Rhodobacteraceae* and *Sphingomonadaceae*; Rho, >0.8), respectively, were enriched in the monocultured hybrid (“moH,” log_10_LDA of >2) (Fig. 3J). While, some pathways, including “transcription machinery” (genetic information processing) and “Arginine and proline metabolism,” with both being attributed to *Brevinemataceae* (Rho = 0.5 and 0.6), were enriched in the monocultured purebreds (moP, log_10_LDA of >3) (Fig. 3J).

### Ecological processes governing pufferfish gut microbiota assemblage in host genetics-environmental interactions.

**FIG 4:**
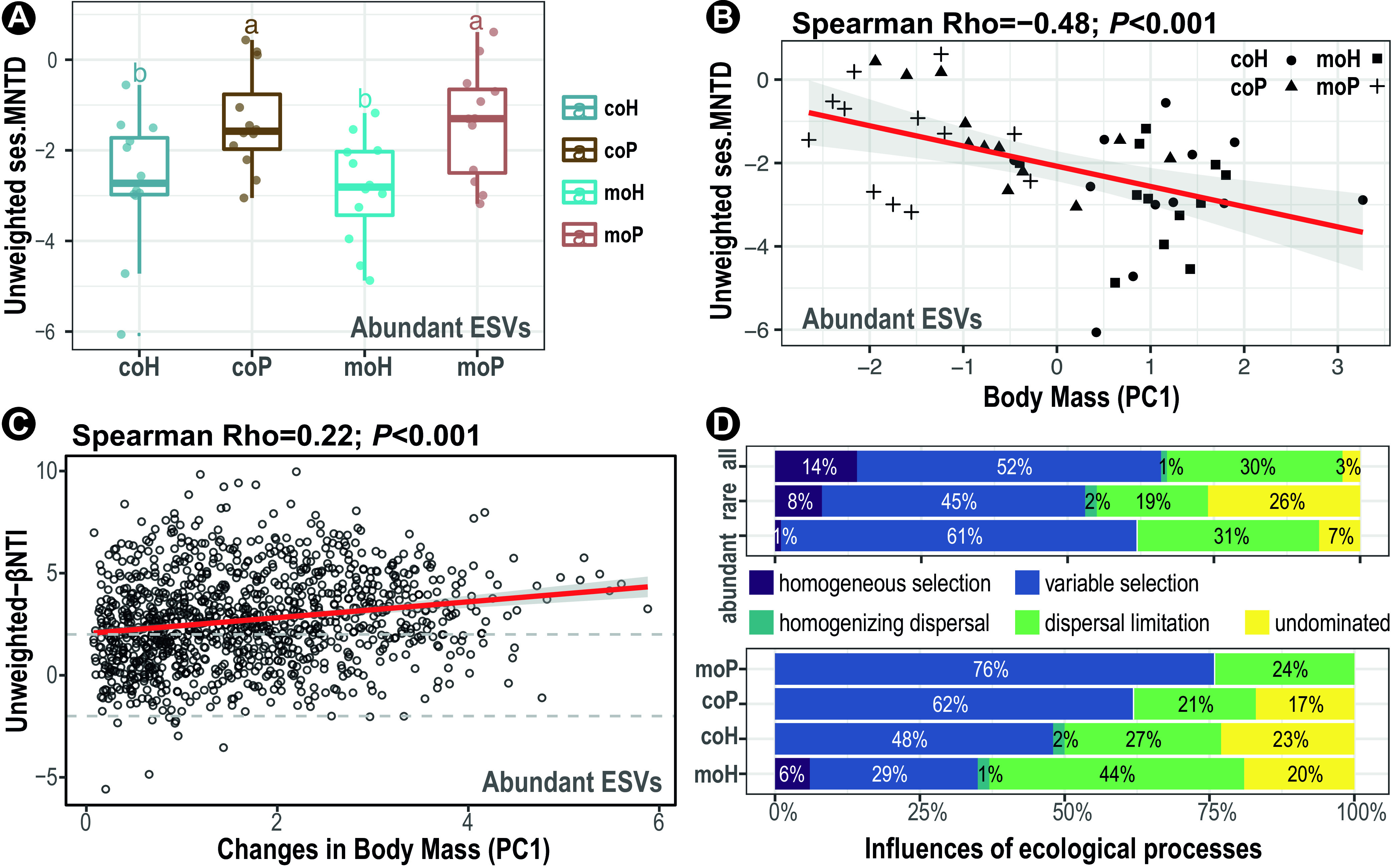
Quantification of ecological processes in governing microbial community assemblage in pufferfish gut. (A) Boxplots denote differences of unweighted-standardized effect size of mean nearest taxon distances (ses.MNTD) across pufferfish groups. Lowercase letters denote statistical significance reported by Mann-Whitney U test at a confidence level of 0.95. (B) Scatterplots show the anticorrelations between unweighted ses.MNTD metrics of the indicated subcommunity and the body mass index (PC1). The red line denotes the linear regression of ses.MNTD and pufferfish body mass (Spearman’s rank). (C) The correlations between phylogenetic beta diversity βNTI and differences in body mass indexes (PC1) in pufferfish gut microbiota for the abundant ESV subcommunity. Red lines denote fitting of linear regressions. Gray horizontal dashed lines denote the cutoff of βNTI significance ranging from −2 to 2. (D) The top three stacked bars depict the relative importance of different ecological processes in governing microbial community assemblage for “all,” “rare,” and “abundant” subcommunities, respectively. And the bottom four stacked bars depict the “abundant” subcommunities of each indicated pufferfish group. Percent font colors, white and black, within the stacked bars denote determinacy and stochasticity fractions, respectively. All statistics were shown as subpanel titles.

The phylogenetic signals of gut microbiota were determined by Blomberg’s K statistic and Mantel’s correlogram to quantify the underlying ecological processes governing microbial community assembly. A positive correlation between phylogenetic signal with a difference of fish body mass (PC1) was observed (*P *< 0.05), and yet it was merely within a narrow range of phylogenetic distance, rendering the quantification of phylogenetic turnover among closely related metataxa applicable to our data set. To detect ecological influences of various importance on submetacommunities that differed in relative abundance (0.01% cutoff), the gut microbiota (“all,” 4,908 ESVs) were fractionated into “abundant” (>0.01%, 326 ESVs) and “rare’ (<0.01%, 4,582 ESVs) submetacommunities. The calculated unweighted-standardized effect size of the mean nearest taxon distances (ses.MNTD) were well below zero for each of these submetacommunities per fish group (*P *< 0.01) (see Fig. S7 in the supplemental material), indicating the importance of environmental filtering in driving phylogenetic clustering of the gut metacommunity. Notably, the abundant submetacommunity in hybrid groups had a greater absolute unweighted ses.MNTD than that in the purebred counterparts (Fig. 4A), and such differences were anticorrelated with the host body mass (Fig. 4B), suggesting a lower degree of phylogenetic relatedness in the gut microbiota of the slow-growth purebred.

10.1128/msystems.01181-22.7 FIG S7 Boxplots denote differences of unweighted-standardized effect size of the mean nearest taxon distances (ses.MNTD) of all (A1), abundant (B1), and rare (C1) subcommunities across pufferfish groups. Lowercase letters denote statistical significance reported by Mann-Whitney U test at a confidence level of 0.95. Scatterplots show the correlations between unweighted ses.MNTD metrics of all (A2), abundant (B2), and rare subcommunities and the body mass index (PC1) (C2). Download FIG S7, PDF file, 0.7 MB. Copyright © 2023 Jin et al. 2023 Jin et al. https://creativecommons.org/licenses/by/4.0/ This content is distributed under the terms of the Creative Commons Attribution 4.0 International license.

Furthermore, the pairwise phylogenetic beta-diversity (βMNTD) was estimated and compared against the null model to calculate the unweighted-beta nearest taxon index, βNTI, of which the absolute values greater than 2 denote significant phylogenetic turnover comparing with those of the randomized community. In addition, results of the Mantel’s test showed significant correlations between βNTI and changes of body mass for each of the submetacommunities grouped by abundance, particularly for the abundant ones (Fig. 4C; see Fig. S8 in the supplemental material). These results demonstrated that the gut microbiota assemblage was attributed to the shift from a small fraction of homogeneous selection (1~14%) to a great majority of variable selection (45 to ~61%) as the changes of host body mass increased, regardless of abundance (Fig. 4D, top). Regarding the stochastic ecological processes with an absolute βNTI value less than 2, their pairwise distances were further partitioned by capping absolute values of both βNTI and RC_bc_ at 2 and 0.95, respectively. The results showed that dispersal limitation (19 to 31%) and “undominated” (3 to 26%) processes contributed largely to nondeterministic ecological processes for each subcommunity grouped by abundance, while the homogenizing dispersal had little to no contribution (0 to 2%) (upper panel in Fig. 4D).

10.1128/msystems.01181-22.8 FIG S8 The correlations between βNTI and differences in body mass (PC1) in pufferfish gut microbiota for all ESVs (A), abundant ESVs (B) and rare subcommunities (C). Red lines denote the fit of linear regressions, and corresponding Spearman’s rank correlation coefficients were indicated in the upper right corner within each subpanel. Gray horizontal dashed lines denote the cutoffs of βNTI significance between −2 and 2. Download FIG S8, PDF file, 2.8 MB. Copyright © 2023 Jin et al. 2023 Jin et al. https://creativecommons.org/licenses/by/4.0/ This content is distributed under the terms of the Creative Commons Attribution 4.0 International license.

Given that the phylogenetic signals of abundant subcommunities were significantly different between purebreds and hybrids (Fig. 4A), the influence of ecological processes determining the assembly of abundant subcommunities was further estimated per fish group. The results showed that the ratio between deterministic and stochastic processes was near six times larger in monoculture purebreds (76%/24%) than that in monoculture hybrids (35%/65%) (Fig. 4D, bottom). Moreover, such differences diminished slightly in cocultures, as evidenced by the decreased and increased proportions of variable selection in monoculture purebreds (from 76% to 62%) and monoculture hybrids (from 29% to 48%), respectively. Consistently, neutral modeling indicated greater deviations in purebred groups (‘coP’: R^2^ = 0.61; ‘moP’: R^2^ = 0.48) than those in hybrid groups (‘coH’: R^2^ = 0.70; ‘moH’: R^2^ = 0.69) while considering all metataxa (see Fig. S9 in the supplemental material).

10.1128/msystems.01181-22.9 FIG S9 Fitness of the neutral model for monocultured purebred (A), cocultured purebred (B), monocultured hybrid (C), and cocultured hybrid (D). Neutral (fit), overrepresented, and underrepresented ESVs are colored gray, red, and blue, respectively. The solid black line denotes model prediction, and the dashed black lines denote the lower and upper bounds of the confidence envelop (0.95). The fitness of the neutral model (R^2^) and migration rate (*m*) are shown as the plot title. Download FIG S9, PDF file, 0.4 MB. Copyright © 2023 Jin et al. 2023 Jin et al. https://creativecommons.org/licenses/by/4.0/ This content is distributed under the terms of the Creative Commons Attribution 4.0 International license.

## DISCUSSION

The main objective of this study was to explore the impacts of host genetics, environment, and interactions between them on pufferfish growth and gut microbiota. Here, the gut microbiota of artificial F1 hybrid pufferfish with marked growth heterosis, along with their maternal halfsibling purebreds, were profiled by 16S rRNA amplicon sequencing, and the underlying interrelations of gut microbiota, host body mass, and fish culture modes were determined. Our results showed that the gut microbiota are compositionally and functionally distinct by host genealogy. Such differences of host growth and gut microbiota both were slightly and yet significantly altered in hybrid-purebred cohabitants, compared with respective monoculture cohorts that shared common genetic make-ups. Furthermore, the phenotypic and functional differences and the ecological processes governing community assemblage of gut microbiota were inferred. Our results not only can enrich, from a microbial perspective, the sophisticated understanding of the complex and dynamic assemblage of fish holobionts but also will provide deeper practical implications for microbial contributions for optimizing performance prediction and improving the production of farmed fishes.

### Effects of hybridization on pufferfish holobiont: implications of host metabolic priority shift toward carbohydrate utilization.

Often, interspecific hybridization can give rise to progeny with enhanced or diminished viability and/or fertility, thereby conferring fitness superiority or inferiority, respectively, relative to parents (19, 44). Here, the F1 hybrid pufferfishes exhibited superior growth performance and intact hypotonic acclimation capacity relative to their maternal halfsibling purebreds (Fig. 1), indicating enhanced fitness associated with intrageneric hybridity. Furthermore, this hybrid cohort can produce viable F2 progeny by backcrossing with the maternal parents equally well as the purebreds (data not shown), suggesting an unimpaired fertility. Considering the husbandry condition, pond management and feeding regime all were identical (within cocultures) or nearly so (between monocultures) for the fish tested here, and the above results indicated strongly that the intrageneric hybridity, over the environment if not entirely, confers growth vigor without apparent fertility cost. In fact, natural hybridization between a given pair of *Takifugu* spp. in the estuarine and near-shore habitats had been reported previously (30, 45, 46), demonstrating an ecologically and behaviorally incomplete prezygotic reproductive isolation thereof. Importantly, *Takifugu* spp. exhibit a relatively lower degree of intrageneric genetic differentiation (i.e., less genetically incompatible), as is evident by its well-conserved karyotype and synteny (46–48), rendering possible the postzygotic synapsis, recombination, and segregation of chromosomes in hybrid progenies. Moreover, artificial hybridization in *Takifugu* had been proven feasible and amendable (39, 43), justifying its promising application potential in breeding practice. Although population genetics had revealed that clear genomic divergence correlated with hypotonic adaptation of *T. obscurus* (33) and fast growth of *T. rubripes* (31), compared with other *Takifugu* spp. with respective lower trait values, the underlying genetics mechanisms of heterosis warrant finer-scale investigations. One plausible explanation for such growth vigor is paternal effects, as is evident by the comparably higher levels of growth hormones in both hybrid progeny (*T. obscurus* ♀ x *T. rubripes* ♂) and male parent (*T. rubripes*) than those in the female parent (*T. obscurus*) (43). Nevertheless, it remains elusive whether such heterosis is conferred by the allele-specific overdominance and/or by the genome-wide heterozygosity *per se*.

In addition to the extrinsic environmental factors, the hybrid fitness can be determined by the compatibilities and interactions, not only between the allospecific host DNA that have never before coadapted and coevolved together (discussed above) but also between their respective symbiotic microbiota that assembled and succeeded independently (49–51). Previously, we showed the *T. obscurus* gut microbiota was replete with copiotrophic bacteria (with higher numbers of rRNA genomic copies) that can proliferate faster, in contrast to the oligotrophic ones predominated in pond water and sediment, indicating a substantial niche barrier between pufferfish gut (nutrient rich) and the surrounding environment (nutrient poor) (38). In addition, three bacterial families, namely, *Brevinemataceae*, *Rikenellaceae*, and *Mycoplasmataceae*, were characterized to be autochthonous (core) pufferfish gut microbiota (38). Consistently, these three bacterial taxa, albeit varied in abundance, were also present in the gut microbiota tested here, regardless of host genealogy (Fig. 2F). Moreover, the hybrids did have a markedly different gut microbiota, as evidenced by a lower alpha diversity (species richness, Fig. 2A and B), higher beta diversity (community dissimilarity, Fig. 2C and D), disproportionate bacterial phenotypes and functional compositions (Fig. 3A, B, E, and J), higher phylogenetic relatedness (ses.MNTD, Fig. 4A and B), and more stochasticity influenced (Fig. 4D) than the purebreds. These results together with our previous findings indicated an evident host genetics-associated niche effect on the gut microbiota, which can be scaled up from the host genus (38) down to the species level (this study). Such a linkage between host genetics and gut microbiota is, albeit to a limited extent, reminiscent of the phylosymbiosis concept, which suggests that more phylogenetically related hosts tend to have more compositionally similar microbiota (8). Mounting evidence has emphasized the importance of both vertical and horizontal transmissions of microbiota in shaping symbiosis, which are underpinned by microbial acquisition and cultivation on or in the host (7, 8). Considering a shared maternal source and hatching environment of the fish tested here, and almost negligible albeit interspecifically different contribution of paternal gamete in early life microbiota exposure thereof, the common microbiota between the hybrid and purebred may signify vertical transmission from the shared maternal parents. While such parent-to-progeny transmission is highly unlikely as faithful as seen in mammals (7, 8), given the proneness to environmental disturbance of fish external fertilization. Conversely, fish gut microbiota can be recurrently sourced from the environment by horizontal transmission, which is determined by the gut physiochemical condition defined by host genetics (21) and/or by the microbial propensity for colonization and proliferation in a given gut environment (40, 41).

Here, the pufferfish gut niches were generally characterized to be favored by the Gram-negative, anaerobic, and biofilm-forming bacteria, wherein the hybrid groups appeared to be more favored by the Gram-positive and facultatively anaerobic bacteria than the purebred ones, as indicated by Bugbase inference (Fig. 3A to I). Moreover, several carbohydrate metabolism-related pathways of the predicted gut metagenomes were enriched in the hybrid groups, which contrasted with the enriched lipid and amino acid metabolism-related pathways in the purebred groups (Fig. 3J). Previous studies revealed higher growth hormone levels in hybrid pufferfish (*T. obscurus* ♀ x *T. rubripes* ♂) than those in purebreds (*T. obscurus*), which can accelerate feed intake and feed efficiency and impact digestive metabolism of feed intake (43). In fact, growth hormone transgenesis in diverse fish species can cause a shift in host metabolic priority, wherein the dietary carbohydrate was used as a preferential energy source over protein and lipid, and its carbohydrate utilization was elevated to meet the high energy demand and spared for fast growth-associated biosynthesis of the protein and lipid (52–54). As such, we speculated that the observed metataxonomic and functional differences of gut microbiota likely reflect the disparate metabolic priorities between pufferfish hosts that differed in genealogy. Considering a likely increased feed intake and energy demand of the hybrid pufferfish tested here, more dietary carbohydrate and hydrolysis intermediates in the gut digesta could be expected, thereby fueling the growth of restricted bacterial taxa specialized for carbohydrate fermentation, such as *Fusobacteriaceae* and *Mycoplasmataceae* (i.e., taxa with high abundance). This point can be further strengthened by the positive correlation between the abundance of the “carbohydrate digestion and absorption” pathway and fish body mass (Fig. 3J; Fig. S6), which can be observed more obviously in hybrid pufferfishes. Conversely, in the absence of a high growth hormone level as in the hybrid, the basic catabolic processes of dietary protein and lipid may have been maintained in the purebred pufferfish gut, from which some catabolites, including essential amino and lipid acids and derivatives, can be made available to and be extensively used by a wider range of microbes as growth factors or precursors, thereby nourishing growth and increasing the overall alpha diversity thereof. In turn, the different gut microbiota of hybrid and purebred groups may also alter the gut physiochemical environment, for instance by microbial metabolites, and further enlarge such intercommunity dissimilarity. Altogether, the above results demonstrated a profound influence of artificial intrageneric hybridization on the pufferfish holobiont, which is plausibly associated with a shift in the metabolic priority of the host.

### Influences of cohabitation on the pufferfish holobiont: implications of environmental enrichment for fish.

Practically, by growing different fish species or populations together, polyculture or coculture not only can enrich ecosystem biodiversity and provisioned function (55) but also enhance environmental enrichment by providing mechanical and physiological stimuli to the cultured fish (56). Here, coculturing hybrid and purebred pufferfishes caused changes to both fish growth (Fig. 1) and gut microbiota (Fig. 2), compared with the equally dense monoculture counterparts with the same genetic background. Despite the dispersions due to an insufficient sample size, the increasing tendency of fish growth in the cocultured subset (sub) remained robust, particularly in the purebred group (Fig. 1). By increasing biodiversity, for instance, by complementarily integrating multitrophic or monotrophic aquaculture, polyculture, or coculture can promote fish growth and reduce waste discharge, ultimately expediting the transition toward more environmentally sustainable aquaculture (57). Nevertheless, the fish growth promotion by coculture observed here is less likely as a result of trophic complementation (e.g., feed utilization) since both parental species, i.e., *T. rubripes* and *T. obscurus*, are omnivores (58). Few promising behavioral studies had shown that *T. rubripes* exhibits quicker acclimation in new environment and shorter latency to both feed and novel object, suggesting a distinct proactive behavior that may linked to fast-growth (59). The genetic basis underpinning such proactiveness was found to be controlled by a combination of coadapted multiallelic loci together with small pleiotropic effect (59). Such an intrageneric difference of host behavior would be a likely explanation for the growth enhancement by cohabitation observed here. It is tempting to speculate that, as a way of environmental enrichment, coculturing of hybrid and purebred pufferfishes that differed in stress copying styles or personalities can sufficiently intensify social interactions (56). For instance, by providing sensory stimuli and social novelty and meanwhile maintaining conspecific and/or congeneric familiarity, social interactions can stabilize the social hierarchy of the pufferfish cohabitant and mitigate the stress response that will inhibit activity and feeding, thereby enhancing growth and ultimately optimizing fitness and survival, as is seen in other fish coculture systems (57).

According to the metacommunity theory, the host-associated microbiota does not exist in isolation and permanence but rather can change membership by reciprocally transmitting in and out from other hosts and the environment (7, 8). Here, the purebred-hybrid cohabitants both have significantly less dissimilar microbiota either between or within genetic groups than that of respective monocultures (Fig. 2D), indicating a measurable influence of cohabitation by diminishing interhost dissimilarity. Of note, an inflated dissimilarity dispersion with reduced distance in each community pair, including cohabitants, was also observed (longer interquartile range in Fig. 2D). Such results somehow reflect the augmented interhost heterogeneity of gut microbiota by cohabitation, in a tendency of reducing dissimilarity particularly apparent among cohabitants (i.e., rows coH.coP, coH.coH, and coP.coP). Such homogenizing effects appeared to have a subtle effect on the phylogenetic relatedness of gut microbiota, as indicated by the comparable ses.MNTD indexes between culture modes per genetic group (Fig. 4A). These results suggest that cohabitation does not homogenize microbial communities simply by averaging diversities (richness and similarity) but rather it involves complex interactions within a metacommunity across all hosts and its environment. As aforementioned, the two *Takifugu* spp. discussed here vary considerably in behaviors (59), and this variation may have implications not only for host growth promotion (e.g., by enriched social interactions) but also for community membership changes of microbes (e.g., by in-between transmission). By stimulating host behavioral and physiological changes, animal social interactions had been shown to have multitude effects on the assemblage of symbiotic microbiota (7, 56, 60). According to the social microbiome concept (7), animal microbiota not only rely on the host for resources but also depend on social interactions and networks of the host (particularly group-living animals) to be transmitted. Therefore, proximity and connectivity within a common environment shared by cohabitated animals (in our case hybrid-purebred cohabitants) are expected to probabilistically promote direct (physical) or indirect microbial transmissions (e.g., fecal contamination) and dispersal, ultimately reducing community dissimilarity (7). Importantly, indirect transmission may involve transient environmental persistence that selects for microbes capable of survival as free-living organisms, for instance, conferred by oxygen tolerance, motility, and surface colonization (7, 41). In supporting this notion, the two discriminative taxa *Fusobacteriaceae* and *Mycoplasmataceae* (Fig. 4E and F) in hybrids by either culture modes both have a strict or facultative anaerobic lifestyle and fermentative abilities, indicating a lack of extrahost persistence as seen in other fish species (61). Conversely, among the most prevalent taxa shared by all pufferfishes, *Aeromonadaceae*, *Flavobacteriaceae*, and *Vibrionaceae* (Fig. 3F) are characterized to be aerobic, motile, and surface adhesive, which is consistent with their ubiquity in the aqueous environment and active interhost transmissions (62, 63).

Furthermore, we observed distinct assemblage patterns between the gut microbiota of purebred and hybrid groups within coculture. For instance, the influence of determinacy in the monocultured purebred group was weakened in coculture (from 76% to 62%), which contrasted with that of the hybrid counterparts that were enhanced in coculture compared with monoculture (from 35% to 48%) (Fig. 4D), suggesting slightly converged ecological processes governing community turnover for both hybrid and purebred cohabitants. Among the determinacy, variable selection (βNTI, >2), rather than homogeneous selection (βNTI, <−2), varied from moderate to strong as the body mass differences increased (Fig. 4C), highlighting the importance of augmented spatial environmental heterogeneity in determining gut microbial community assemblage (6, 10). In our case of coculture, such variable selection could be attributed to the enhanced complexities of host-microbial interactions within and/or between hybrid and purebred groups. Given the cohabitants with presumptively different behavior and the spatially and temporally connected gut microbiota, either or both can cause more fluctuations in the gut microenvironment, thereby imposing an enlarged heterogeneous influence on the community composition, as reflected in increased dissimilarity dispersion with reduced dissimilarity within coculture community pairs (Fig. 2D). Moreover, a large fraction of stochastic influence was found governed by dispersal limitation (~30%) and undominated processes (~15%) and little (<1%) to no influence by homogenizing dispersal (Fig. 4D). The undominated fraction can be regarded as consequences of attenuated selection and/or dispersal, with both being the opposite of variable selection (6). In supporting this notion, the undominated fraction is absent only in the gut microbiota of the monocultured purebred group, which is influenced largely by variable selection. Notably, the stochastic influence, particularly dispersal limitation, was found prominent in monoculture hybrids (44%) but less so in cocultured counterparts (27%). In contrast with homogenizing dispersal (stronger dispersal), dispersal limitation indicates the host effect (e.g., as patched niche), by which the magnitude to which microbes transmit among individual gut was constrained (6). Such higher levels of stochastic influence in hybrids, particularly monocultures, may be associated with a lower degree of variations in intra- and/or extrahost environments, which were implicated with prioritized metabolism of carbohydrate and a lack of environmental enrichment for the host (discussed above). In line with this notion, a neutral model-based inference suggested a greater importance of passive migration and demographic processes in explaining the compositional turnover for both cocultured and monocultured hybrid groups, as reflected by the higher model fitness (R^2^, ~0.7) and yet slightly lower migration rate (*m*, ~0.05), compared with that of purebred ones (R^2^, ~0.5; *m*, ~0.18). These results stress that weaker dispersal (i.e., higher fractions of dispersal limitation) can cause dissipation within local gut communities in the form of reducing alpha diversity (9), as seen in the hybrid group (Fig. 2A).

In conclusion, we show that the gut microbiota relationships between artificial F1 hybrid and purebred pufferfishes well recapitulated their genealogy, indicating evident host genetic effects on gut microbiota. Furthermore, significant fish growth promotion and gut microbiota alteration mediated by hybrid-purebred cohabitation were observed, in addition to the influence of intrageneric hybridization to a larger extent. The compositional and functional congruence of gut microbiota between host genealogy were likely determined in part by the host metabolic priority shift toward carbohydrate utilization. Moreover, the underlying assemblage mechanisms of gut microbiota were found involved in a trade-off between variable selection and dispersal limitation, which was plausibly driven by augmented social interactions between hybrid and purebred cohabitants with different behaviors. Nevertheless, this study cannot explicitly ascertain whether such host-genetic determination is inheritable in the long run and to what extent and if the hybrid heterosis can remain intergenerationally constant (i.e., without breakdown), considering the lack of parental samples, particularly of brackish water-resident *T. rubripes*, examined in parallel. An important caveat in this study is the lack of replicated culture ponds due to limited space and resources. Future studies with enlarged pond replicates and sample sizes need to account for genetic and ecophysiological aspects of the holobiont that contribute to the host fitness and microbial transmission. Altogether, our findings emphasized the critical interfacial roles of gut microbiota in host genetics-environmental interactions and would have deeper practical implications for microbial contributions to optimize performance prediction and improve the production of farmed fishes.

## MATERIALS AND METHODS

### Experimental design and sampling.

The 180 days posthatch (dph) juvenile pufferfishes were obtained from a single commercial supplier (Jiangsu Zhongyang Group Co., Ltd.) located in Nantong, East China, in September 2020, as described previously (39). In brief, the artificial F1 hybrids (H) were produced by crossing four each of *T. obscurus* (♀ siblings) and *T. rubripes* (♂ siblings), and their halfsibling *T. obscurus* purebreds (P) were produced by the same maternal parents. The hybrids and purebreds were either (i) grouped by the same genealogy and then reared separately (2× monoculture, moH and moP) or (ii) equal proportionally cohabitated (1× coculture, coH and coP), in a total of three adjacent indoor ponds (6 m by 6 m by 1 m) at an equal stocking density (Fig. S1A). The husbandry conditions, pond management, and feeding regime all were controlled to be identical (within cocultures) or nearly so (between monocultures). A sum of 400 individual pufferfishes, more precisely 100 fishes per group, were captured randomly from each pond and were immediately weighted and measured for fork length. For gut microbiota profiling, a subset of 48 individuals (*n* = 12 per groups) from aforementioned fishes were selected randomly and sacrificed. After being surface sterilized using 75% ethanol, the distal intestine (2 cm long) was removed with a sterilized spatula to collect the gut content. Subsequently, all gut content samples were placed in separate sterile tubes, and the feed samples were packaged in airtight sterile plastic bags. All microbial samples were preserved with dry ice during shipment and stored at −80°C. The detailed metadata for each sample were recorded in Table S1. The sampling was performed in line with the protocol approved by the Ethics Committee of Experimental Animals (Hohai University) and was in accordance with the Animal Care Guidelines issued by the Ministry of Science and Technology (China).

### DNA isolation and 16S rRNA amplicon sequencing.

The microbiota of gut contents (*n* = 48) and feed (*n* = 3) were profiled by 16S rRNA amplicon sequencing. In brief, DNA was extracted with the FastDNA spin kit for soil (MP Biomedicals, Irvine, CA) and verified with 0.8% agarose gel electrophoresis. DNA integrity and concentration were evaluated by a Nanodrop ND2000 instrument (Thermo Fisher Scientific Inc., Waltham, MA) and a Quant-iT PicoGreen double-stranded DNA (dsDNA) kit (Thermo Fisher Scientific Inc.), respectively. The V4/V5 hypervariable region was amplified using primer pair 515F (5′-CTGCCAGCMGCCGCGGTAA-3′) and 926R (5′-CCGTCAATTCMTTTRAGTTT-3′) barcoded with unique eight-nucleotide sequences per sample. The 25-μL PCR system included 40 ng template DNA, 5 μL 5× reaction buffer, 5 μL 5× GC buffer, 2 μL dNTP (2.5 mM), 10 pM barcoded primer pairs, and 0.75 U Q_5_ high-fidelity DNA polymerase (New England BioLabs [NEB], Ipswich, MA). All samples were tested in triplicate together with no-template controls. The thermocycling conditions were set at 98°C for 2 min; 25 cycles at 95°C for 15 s, 55°C for 30 s, and 72°C for 30 s; followed by a final step of 72°C for 5 min. Amplicons were subjected to electrophoresis with a 2% agarose gel, were purified by the AMPure XP kit (Beckman Coulter GmbH, USA), and were quality checked by a high-sensitivity DNA kit (Agilent, Santa Carla, CA) and Quant-iT PicoGreen dsDNA kit. Purified amplicon DNA libraries were constructed using a TruSeq Nano DNA LT library prep kit (Illumina, San Diego, CA), and the 400-bp insertion was paired-end sequenced with the MiSeq reagent kit V3 chemistry on an Illumina MiSeq platform.

### Read processing and microbiota profiling.

Raw amplicon reads were trimmed by Trim-Galore (Cutadapt v.1.4.2 and FastQC v.0.10.1) with a default Q20 quality cutoff (64). Trimmed reads were processed according to a pipeline described previously (65) with USEARCH v.10.0 (66). Briefly, paired-end reads were merged by “-fastq_mergepairs”; primer sequences were removed by “-fastx_truncate”; and low-quality and redundant reads (error rates, >0.01) were filtered and dereplicated by “-fastq_filter” and “-fastx_uniques,” respectively. The ESVs were clustered after filtering chimeras by “-unoise3.” Subsequently, the ESV table was generated by “-otutab,” and taxonomically assigned by the sintax algorithm with the Ribosomal Database Project (RDP) classifier (RDP training set v.16) (67). The potential organelle (mitochondria and chloroplast)-derived reads were removed by taxonomy-based filtering. The sequencing depth was normalized by “otutab_norm,” yielding 10,000 sequences per sample. Community diversity was profiled by “-alpha_div,” “-alpha_div_rare,” “-cluster_agg,” and “-beta_div.” To identify the featured taxa among different microbial communities, RandomForest (v.4.6-14) (68) was used to classify the microbial taxa in the family level across different groups with parameters of “ntree = 1000, importance = TRUE, proximity = TRUE,” followed by cross-validation by the *rfcv()* function. For functional inference, the ESV table was normalized according to 16S rRNA copy numbers and annotated by PICRUSt (69) with closed-reference picking against a compatible version of the Greengenes database (v.13_5) (70). Differences in relative KEGG pathway abundance were identified with a cutoff of logarithmic LDA score greater than 2 combined with the linear discriminant analysis effect size (LEfSe) algorithm (71). Bacterial phenotypes, such as oxygen tolerance, Gram staining, and pathogenic potential, within each indicated microbial community were inferred by BugBase (72) with the taxonomic level set at bacterial family.

### Quantifying ecological processes for governing microbial community assemblage.

The tree distance-based phylogenetic relatedness inference of a given group of gut microbial community was calculated by *phyloSignal()* and *phyloCorrelogram()* within the “Phylosignal” package (73). The standardized effect size of the mean nearest taxon distances (ses.MNTD) was calculated by the *ses.mntd()* function within the “Picante” package (74) per community, with options of “taxa.labels” as the null expected model, unweighted, and 999 randomization. The ses.MNTD that were significant less than zero indicate closer phylogenetic relatedness than expected by chance (75). To further quantify the relative importance of ecological processes in governing assemblage in microbial community, both metrics of β-nearest taxon index (βNTI) and Bray-Curtis-based Raup-Crick (RC_BC_) were calculated to evaluate the contributions of determinacy and stochasticity, respectively (6). In doing so, the phylogenetic beta diversity was calculated based on unweighted intercommunity MNTD metric (βMNTD) by the *comdistnt()* function. Thereafter, the deviation of observed βMNTD from the null expectation (999 randomization), i.e., βNTI, was computed as described previously (6). The influence of determinacy, including homogeneous selection and variable selection, was inferred with significance βNTI that less than −2 and greater than 2 (6). Subsequently, the nonsignificant fractions that have an |βNTI| value less than 2, reflective of stochasticity, were further subdivided according to β-diversity metrics of RC_BC_, wherein the RC_BC_ indexes normalize the deviation of observed Bray-Curtis from null expectation (9,999 randomization) into a range between −1 and 1. The significant RC_BC_ indexes that are less than −0.95 or greater than 0.95 were interpreted as influenced by homogenizing dispersal and dispersal limitation, respectively. The nonsignificant fractions (i.e., |RC_BC_| of <0.95) were considered community assemblage influenced by undominated processes without a main effect by any given ecological pressure (i.e., weak dispersal and/or selection).

### Statistical analysis.

All statistical analyses were conducted under the R environment (v.3.6.0). Prior to the analysis, the normality of residuals and homogeneity of variances were tested by *shapiro.test()* and *bartlett.test()*, respectively, followed by parametric or nonparametric tests. In doing so, the group means of parametric data sets were compared by one-way and/or two-way (i.e., plus asterisk between two explanatory variables in the formula to account for potential interactions) analysis of variance (ANOVA) using *aov()*, followed by *post hoc* test by *TukeyHSD()*. Alternatively, the rank sums of nonparametric data sets were compared by Kruskal-Wallis test with *kruskal.test()*, followed by Wilcoxon rank-sum test by *wilcox_test()*. The two-way nonparametric statistical tests were performed after the ART procedure by *art()* within the “ARTool” package, followed by model fit test by *anova().* Statistical significance was considered while reported false discovery rate (FDR)-adjusted *P* values were less than 0.05. The PERMDISP test was used to evaluate the homogeneity of multivariate dispersion of groups by *betadisper()*, followed by permutation test with *permutest()*. Principle-coordinate analyses (PCoAs) were conducted based on the Jaccard dissimilarity metric by *cmdscale()*, followed by the permutational multivariate analyses of variance (PERMANOVA) test on the model considering fish body mass (PC1) as a covariate plus the interactions between host genetics and culture mode by *adonis()* within “vegan” package (76). The clustering analysis on fish body mass was visualized after principal-component analysis by *prcomp()*. Potential correlations between βNTI and indicated trait difference (Euclidean-based trait distance matrix) were assessed by *mantel()* within the “ecodist” package (77) with 9,999 permutations. The fitness of the neutral model (78) on the gut microbial community was evaluated by *neutral.fit()* within the “MicEco” package.

### Data availability.

All raw reads generated from this study were deposited at the National Center for Biotechnology Information (NCBI) sequencing reads archive (SRA), under BioProject accession number PRJNA841699.
