# Supplementary figures and images for: Host Hybridization Dominates over Cohabitation in Affecting Gut Microbiota of Intrageneric Hybrid Takifugu Pufferfish
Source: mSystems. 2023 Feb 23;8(2):e01181-22. doi: 10.1128/msystems.01181-22 (PMC10134855; doi:10.1128/msystems.01181-22)

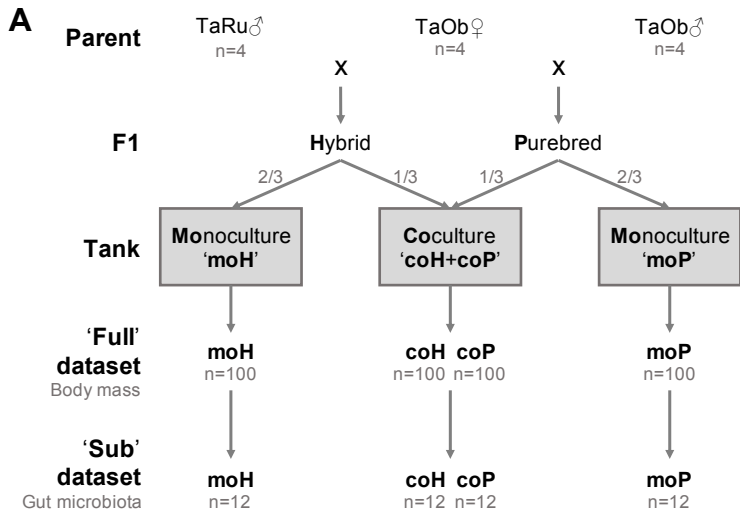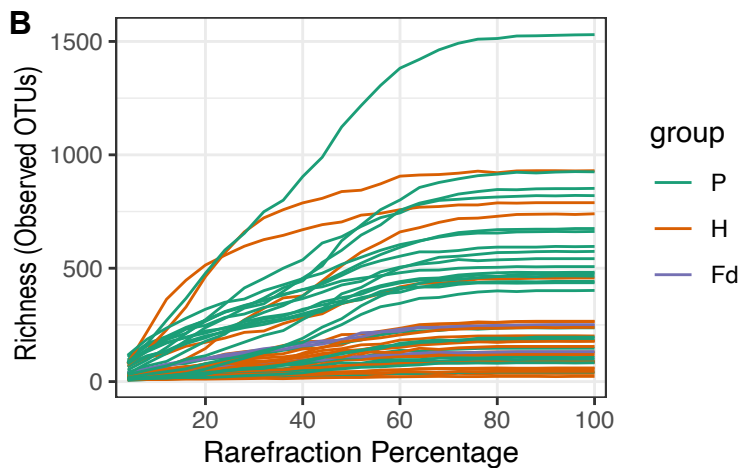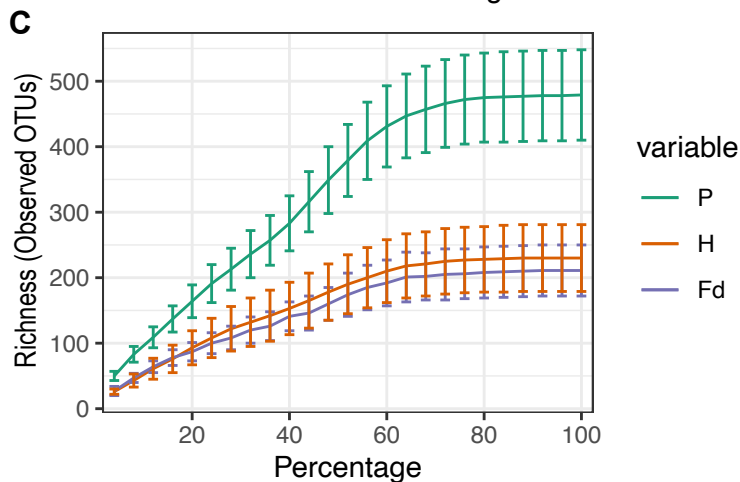

Supplement: FIG S1 [file msystems.01181-22-s0001.pdf]

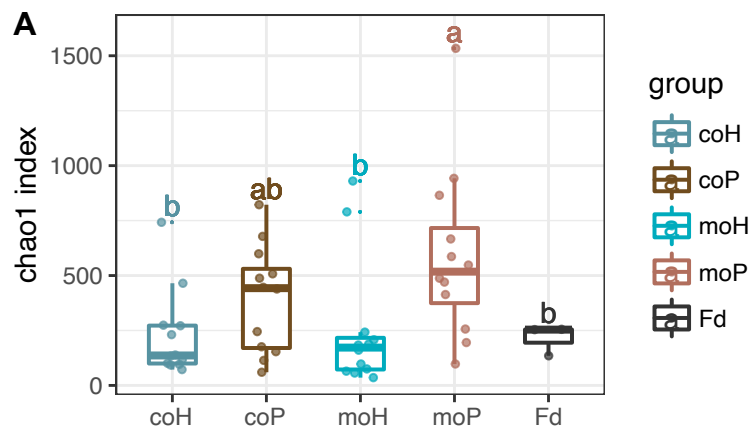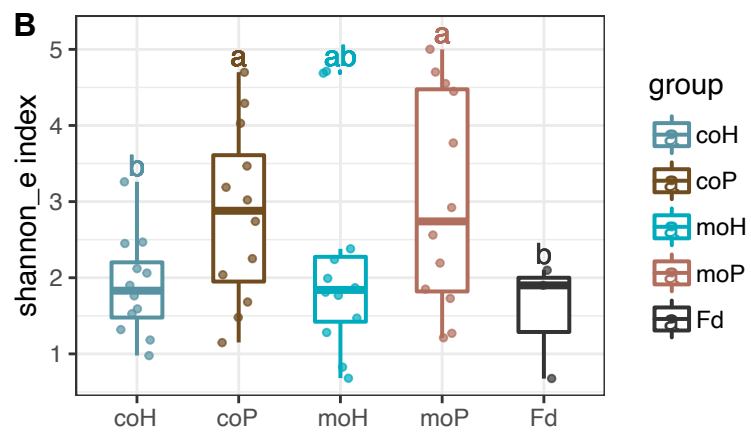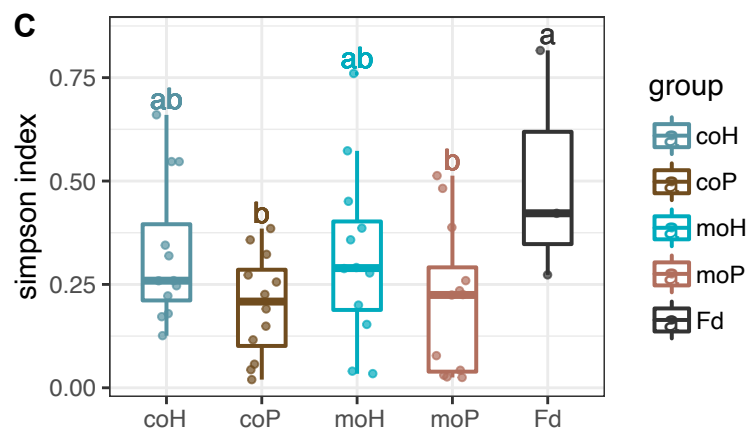

Supplement: FIG S2 [file msystems.01181-22-s0002.pdf]

**A** PERMDISP

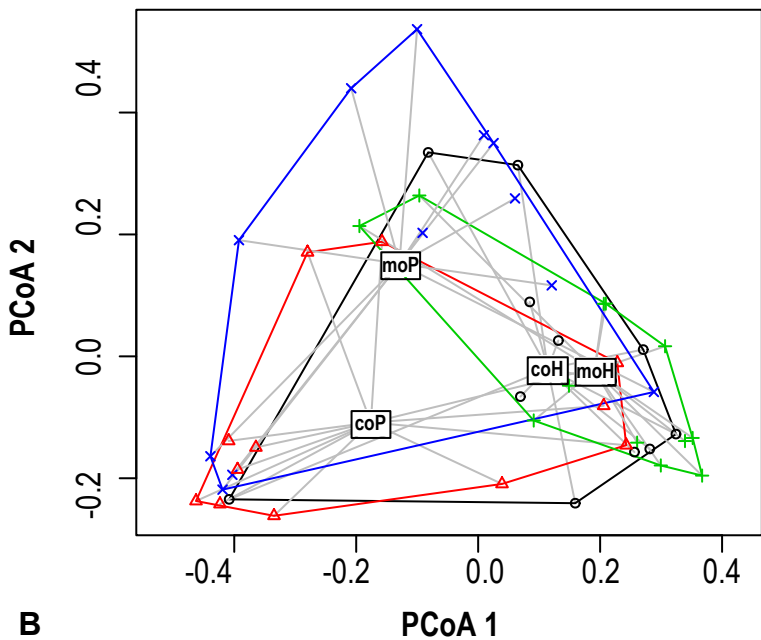

**B**

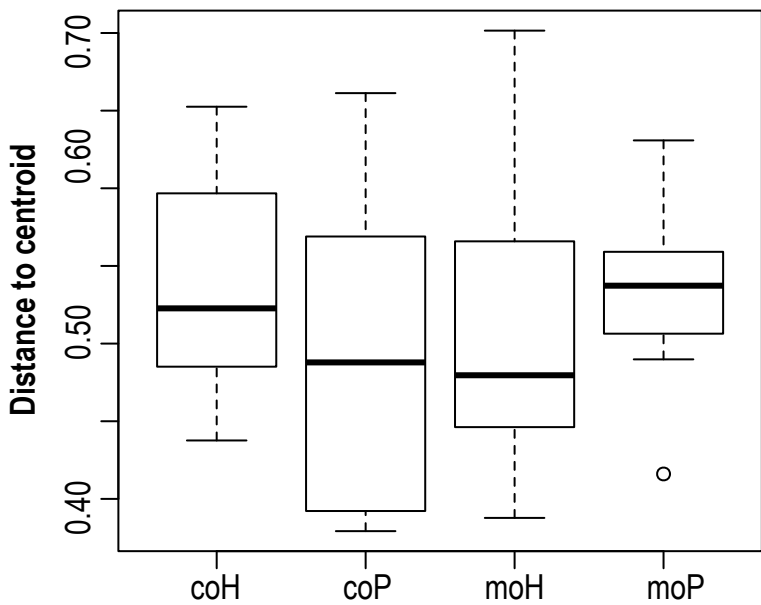

Supplement: FIG S3 [file msystems.01181-22-s0003.pdf]

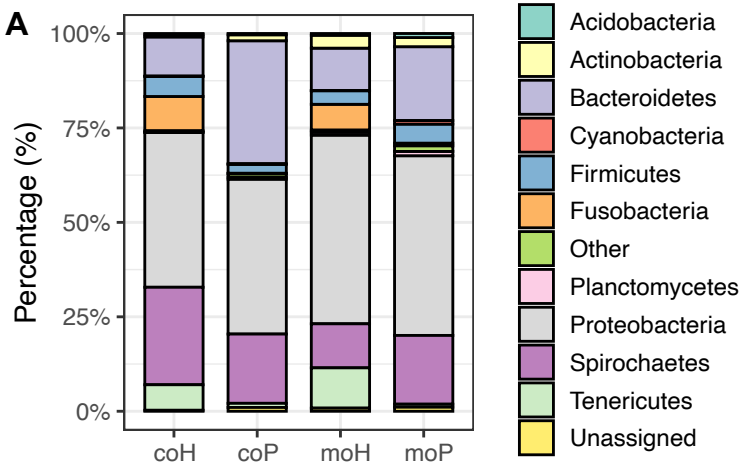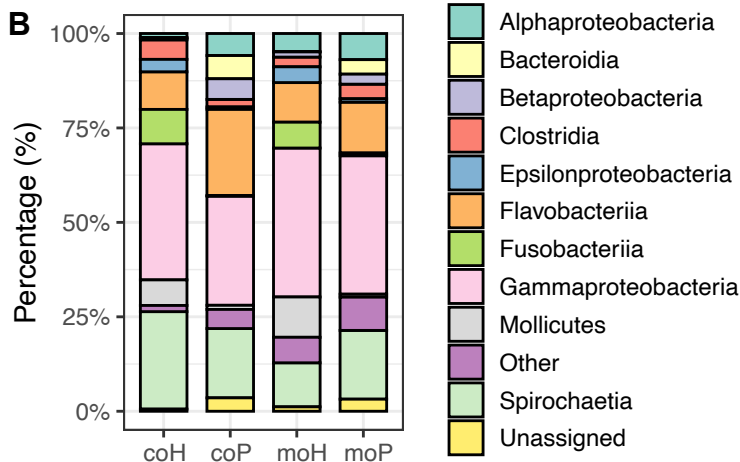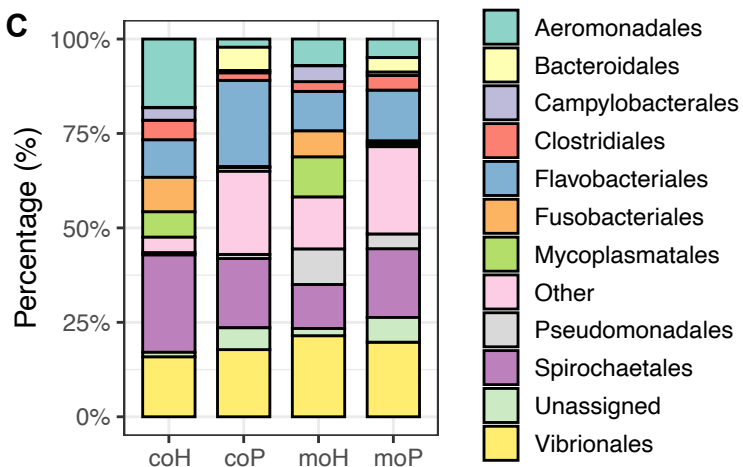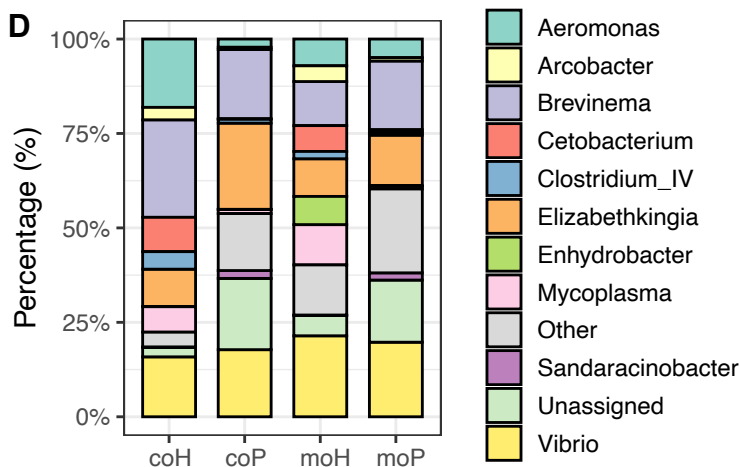

Supplement: FIG S4 [file msystems.01181-22-s0004.pdf]

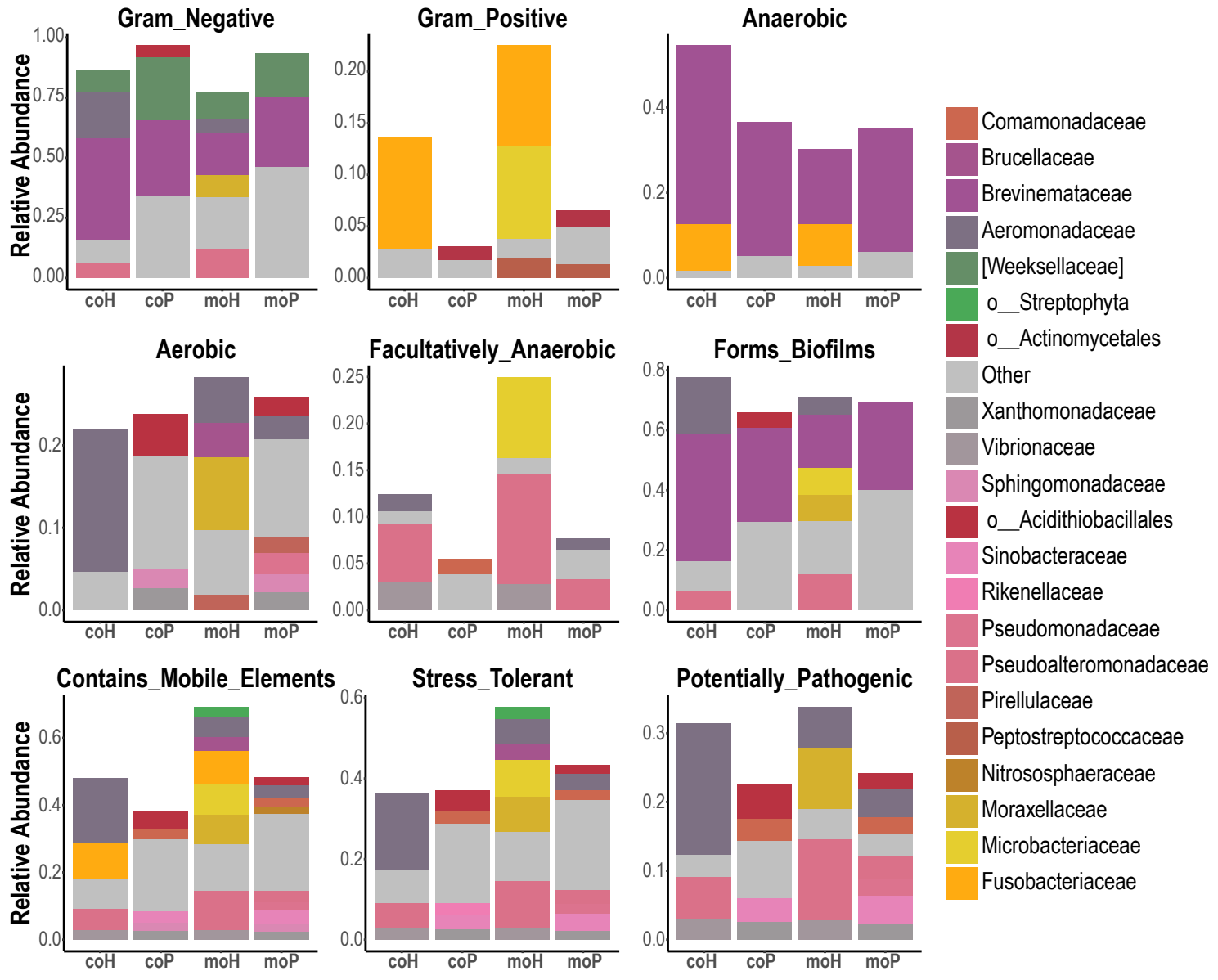

Supplement: FIG S5 [file msystems.01181-22-s0005.pdf]

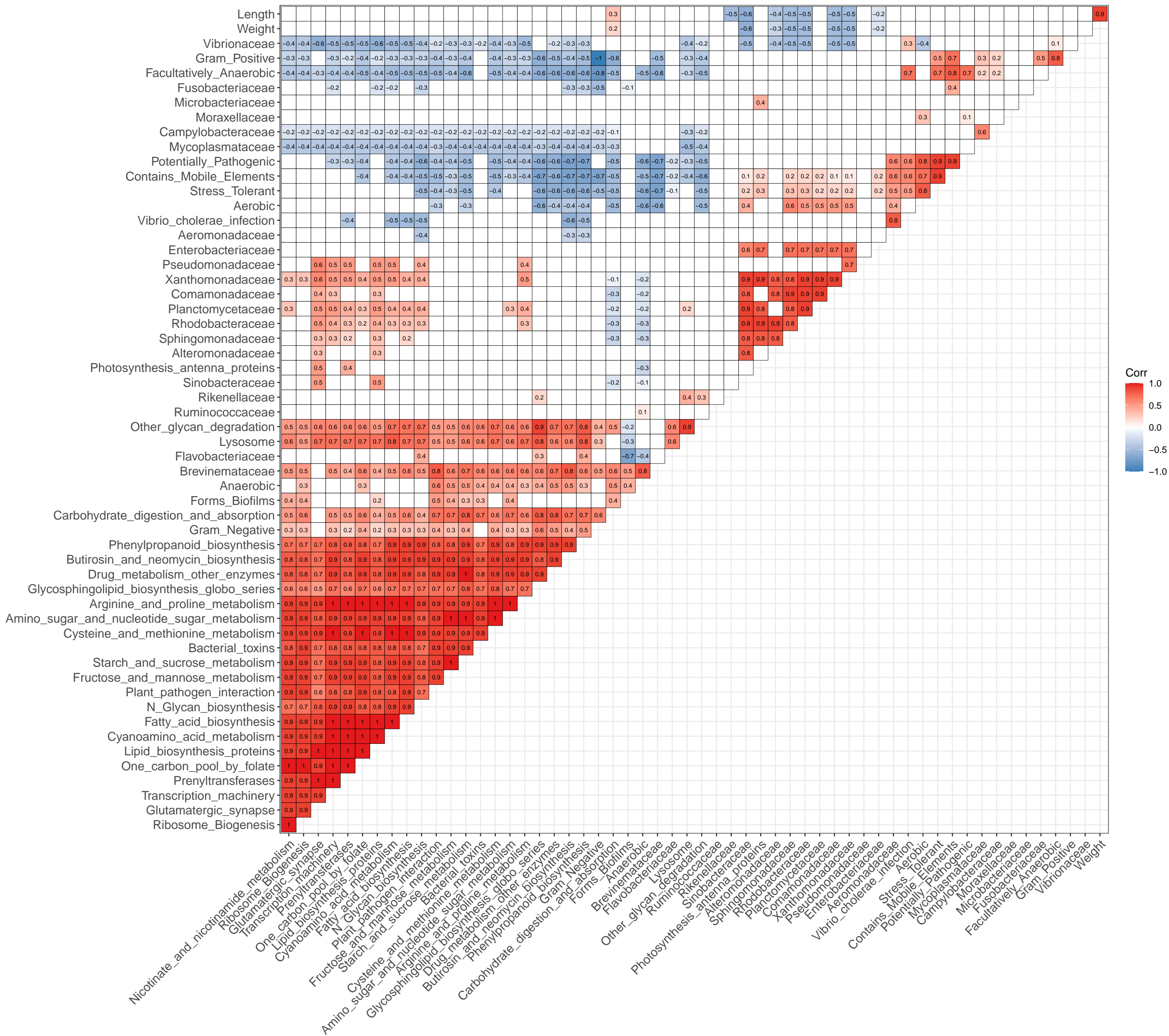

Supplement: FIG S6 [file msystems.01181-22-s0006.pdf]

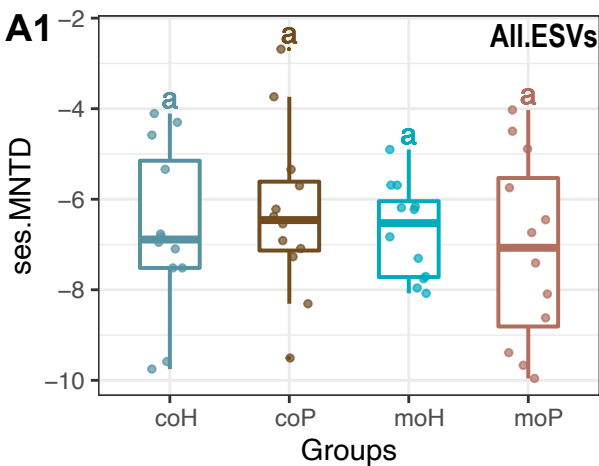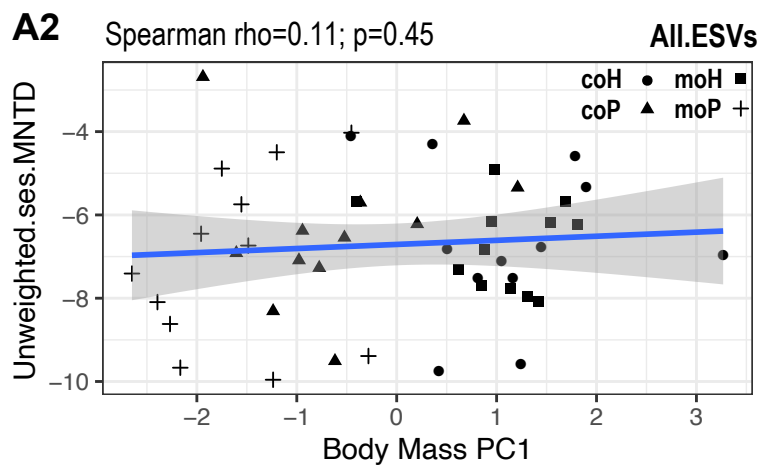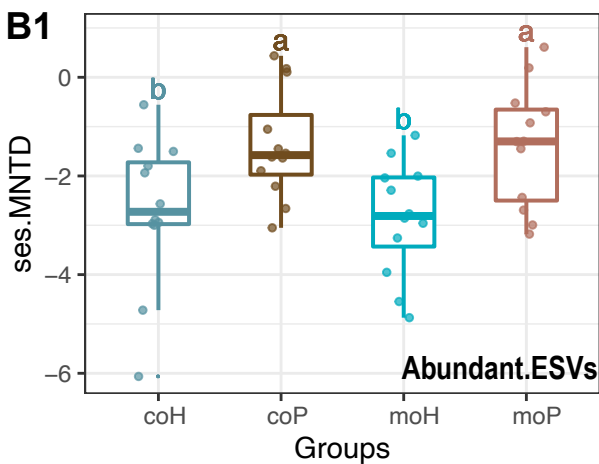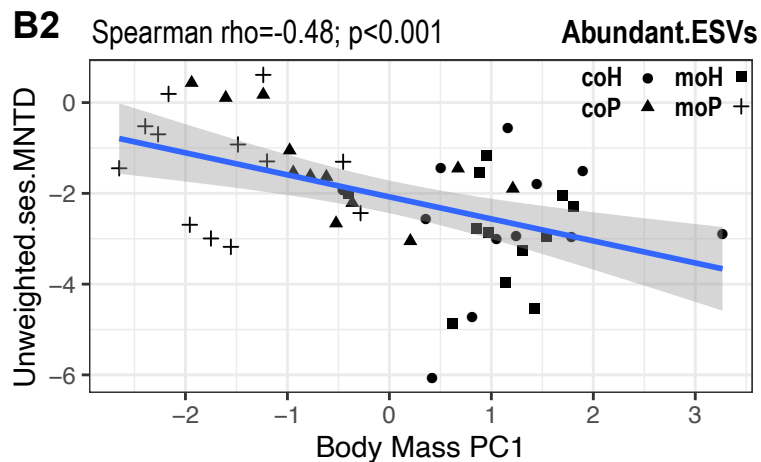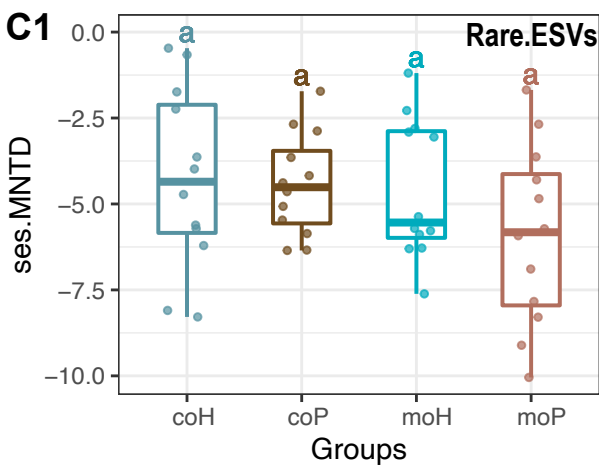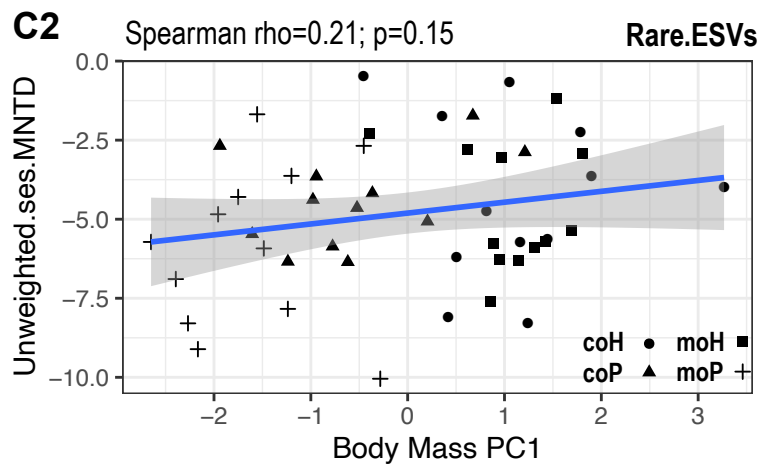

Supplement: FIG S7 [file msystems.01181-22-s0007.pdf]

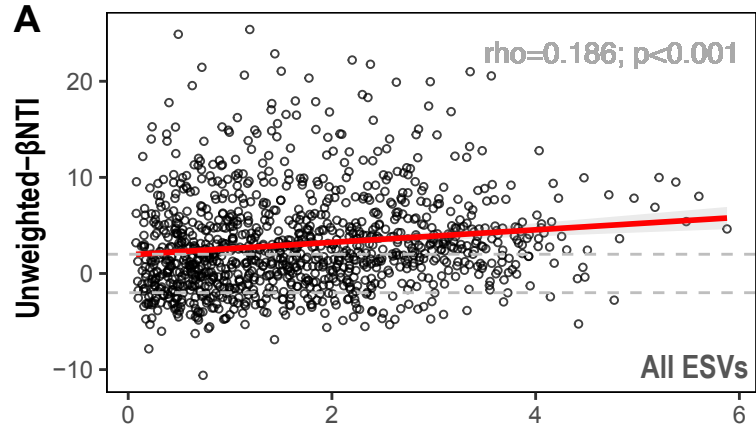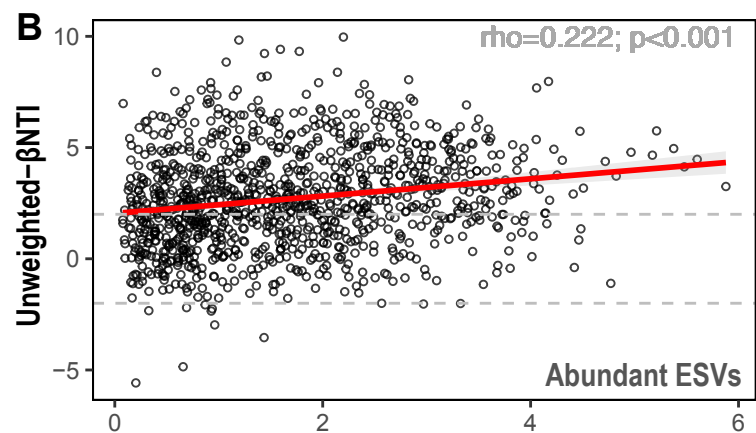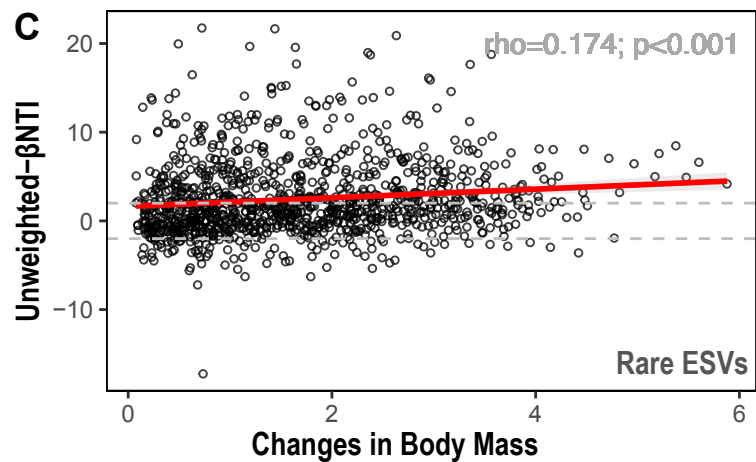

Supplement: FIG S8 [file msystems.01181-22-s0008.pdf]

**A**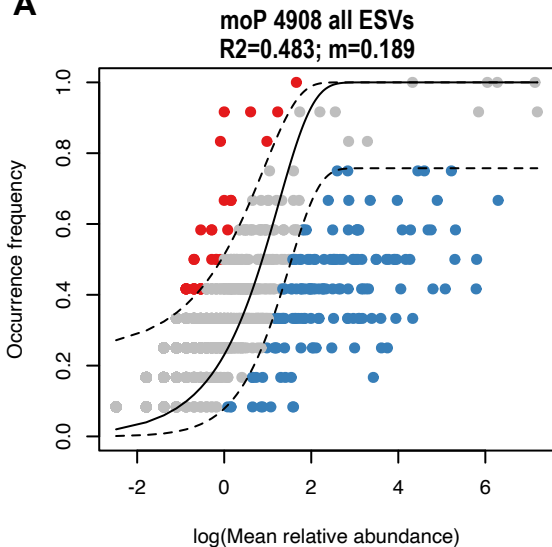**B**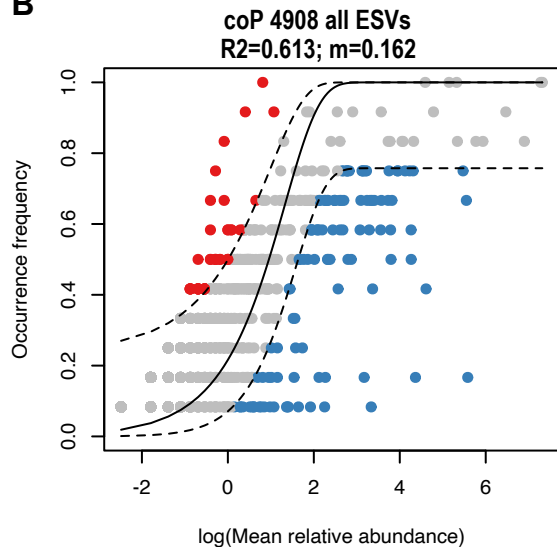**C**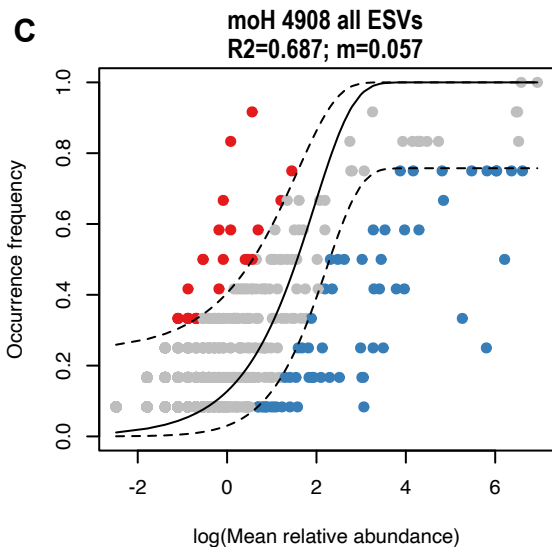**D**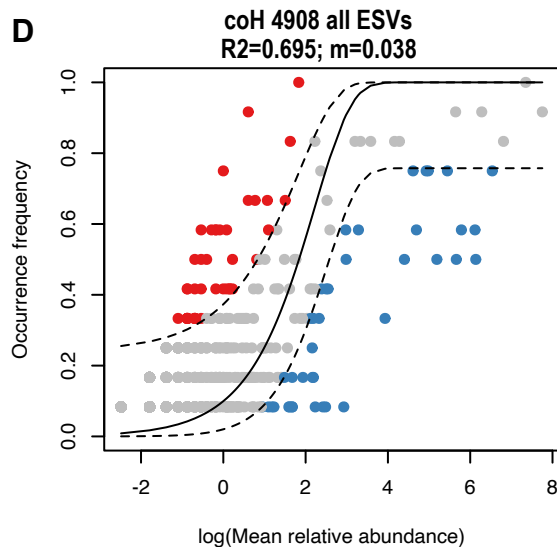

Supplement: FIG S9 [file msystems.01181-22-s0009.pdf]
