# Supplementary material for: Host Hybridization Dominates over Cohabitation in Affecting Gut Microbiota of Intrageneric Hybrid Takifugu Pufferfish
Source: mSystems. 2023 Feb 23;8(2):e01181-22. doi: 10.1128/msystems.01181-22 (PMC10134855; doi:10.1128/msystems.01181-22)
Supplement: TABLE S1 [file msystems.01181-22-s0010.pdf]

Table.1 Metadata.

coH	Weight	Length	PC1	PC2	Reads Size
coH1	61.5	13.2	0.42	0.08	117532
coH2	67.43	13.7	1.05	0.16	124206
coH3	74.24	14.5	1.90	0.14	162996
coH4	62.59	13.8	0.81	-0.18	121266
coH5	65.66	14.1	1.16	-0.16	118499
coH6	52.38	12.6	-0.46	-0.14	129290
coH7	83.37	16	3.27	-0.13	120553
coH8	67.9	14	1.24	0.03	110461
coH9	68.55	14.3	1.45	-0.10	136425
coH10	59.3	13.6	0.51	-0.27	110893
coH11	62.28	13	0.36	0.24	111584
coH12	67.87	15	1.79	-0.52	127247
coP1	44.2	11.4	-1.61	0.02	114417
coP2	53.92	12.6	-0.36	-0.05	94186
coP3	51.56	11.8	-0.94	0.24	94103
coP4	51.88	11.7	-0.98	0.32	98375
coP5	49.4	11.5	-1.24	0.28	95872
coP6	46.8	10.5	-1.94	0.67	114878
coP7	65.55	14.2	1.21	-0.22	122473
coP8	53.33	12.2	-0.62	0.13	113084
coP9	59.74	13	0.21	0.08	123740
coP10	52.53	12	-0.78	0.19	58520
coP11	54	12.3	-0.52	0.12	114320
coP12	62.07	13.6	0.67	-0.10	120573
moH1	66.68	13.6	0.95	0.17	64642
moH2	72.79	14	1.54	0.32	121179
moH3	67.15	13.9	1.14	0.04	136713
moH4	72.66	14.3	1.69	0.15	119052
moH5	74.5	14.3	1.80	0.26	102976
moH6	65.25	13.8	0.97	-0.02	109744
moH7	65.06	13.6	0.85	0.08	111680
moH8	59.4	13.8	0.62	-0.37	125831
moH9	66.43	13.5	0.88	0.21	86474
moH10	70.88	14	1.42	0.21	128008
moH11	69	14	1.31	0.10	113454
moH12	51.56	12.8	-0.40	-0.30	108449
moP1	48.82	13	-0.45	-0.58	110890
moP2	43.97	12.1	-1.24	-0.38	79897
moP3	41.07	11.1	-1.96	-0.01	145167
moP4	38.73	10.09	-2.65	0.40	100472
moP5	38.52	11	-2.17	-0.11	109300
moP6	36.54	10.8	-2.39	-0.12	116979
moP7	46.39	11.9	-1.20	-0.12	89958
moP8	40.76	12	-1.49	-0.52	117895
moP9	57.98	12.3	-0.28	0.36	106804
moP10	40.89	11.5	-1.75	-0.24	105793
moP11	36.82	11	-2.27	-0.21	124595
moP12	43.23	11.6	-1.55	-0.15	121270
